# Time-efficient three-dimensional transmural scar assessment provides relevant substrate characterization for ventricular tachycardia features and long-term recurrences in ischemic cardiomyopathy

**DOI:** 10.1038/s41598-021-97399-w

**Published:** 2021-09-28

**Authors:** Susana Merino-Caviedes, Lilian K. Gutierrez, José Manuel Alfonso-Almazán, Santiago Sanz-Estébanez, Lucilio Cordero-Grande, Jorge G. Quintanilla, Javier Sánchez-González, Manuel Marina-Breysse, Carlos Galán-Arriola, Daniel Enríquez-Vázquez, Carlos Torres, Gonzalo Pizarro, Borja Ibáñez, Rafael Peinado, Jose Luis Merino, Julián Pérez-Villacastín, José Jalife, Mariña López-Yunta, Mariano Vázquez, Jazmín Aguado-Sierra, Juan José González-Ferrer, Nicasio Pérez-Castellano, Marcos Martín-Fernández, Carlos Alberola-López, David Filgueiras-Rama

**Affiliations:** 1 University of Valladolid, Laboratorio de Procesado de Imagen, Valladolid, Spain; 2 Centro Nacional de Investigaciones Cardiovasculares (CNIC), Myocardial Pathophysiology Area, Madrid, Spain; 3 Universidad Politécnica de Madrid, Biomedical Image Technologies, ETSI Telecomunicación, Madrid, Spain; 4 Centro de Investigación Biomédica en Red de Bioingeniería, Biomateriales y Nanomedicina (CIBER-BBN), Madrid, Spain; 5 Instituto de Investigación Sanitaria del Hospital Clínico San Carlos (IdISSC), Cardiovascular Institute, Madrid, Spain; 6 Centro de Investigación Biomédica en Red de Enfermedades Cardiovasculares (CIBERCV), Madrid, Spain; 7 Philips Healthcare Iberia, Madrid, Spain; 8 Hospital Ruber Juan Bravo Quironsalud UEM, Cardiology Department, Madrid, Spain; 9 IIS-University Hospital Fundación Jiménez Díaz, Cardiology Department, Madrid, Spain; 10 Hospital Universitario La Paz, Cardiology Department, Madrid, Spain; 11 Fundación Interhospitalaria para la Investigación Cardiovascular (FIC), Madrid, Spain; 12 Barcelona Supercomputing Center (BSC), Barcelona, Spain; 13 ELEM Biotech SL., Barcelona, Spain

**Keywords:** Cardiology, Cardiovascular diseases, Computational biology and bioinformatics, Image processing

## Abstract

Delayed gadolinium-enhanced cardiac magnetic resonance (LGE-CMR) imaging requires novel and time-efficient approaches to characterize the myocardial substrate associated with ventricular arrhythmia in patients with ischemic cardiomyopathy. Using a translational approach in pigs and patients with established myocardial infarction, we tested and validated a novel 3D methodology to assess ventricular scar using custom transmural criteria and a semiautomatic approach to obtain transmural scar maps in ventricular models reconstructed from both 3D-acquired and 3D-upsampled-2D-acquired LGE-CMR images. The results showed that 3D-upsampled models from 2D LGE-CMR images provided a time-efficient alternative to 3D-acquired sequences to assess the myocardial substrate associated with ischemic cardiomyopathy. Scar assessment from 2D-LGE-CMR sequences using 3D-upsampled models was superior to conventional 2D assessment to identify scar sizes associated with the cycle length of spontaneous ventricular tachycardia episodes and long-term ventricular tachycardia recurrences after catheter ablation. This novel methodology may represent an efficient approach in clinical practice after manual or automatic segmentation of myocardial borders in a small number of conventional 2D LGE-CMR slices and automatic scar detection.

## Introduction

Delayed gadolinium-enhanced cardiac magnetic resonance (LGE-CMR) imaging represents a well-established method to identify infarct-related scar tissue based on signal intensity criteria after gadolinium administration^1^. Several studies have shown that scar tissue quantification using LGE-CMR may increase the predictive performance of ventricular arrhythmic events in patients with established myocardial infarction^2,3^. Nonetheless, scar characterization remains controversial and has not been incorporated as a standard stratification tool for ventricular arrhythmic events or ventricular tachycardia (VT) characterization in patients with ischemic cardiomyopathy (ICM)^4^.

Two-dimensional LGE-CMR sequences with large slice thickness (normally 8 mm) are the most common images in the clinic^5^. Conversely, 3D sequences with higher and isotropic resolution are substantially less common in daily clinical practice, although frequently performed in research studies^6^. State-of-art 3D LGE-CMR sequences require larger acquisition time, appropriate patient collaboration, and time-consuming manual or semiautomatic processing for accurate myocardial segmentation^7^. Altogether, these drawbacks make 3D LGE-CMR sequences potentially inefficient in highly demanding agendas during regular clinical practice. The common alternative using 2D sequences provides sufficient information for assessing ventricular function. However, 2D-derived scar reconstructions may have limitations to properly assess the myocardial substrate compared to isotropic high-resolution 3D data. Moreover, these differences might have implications on accurate characterization of the ventricular substrate associated with VT episodes in patients with ICM.

Recently, we have developed a 3D methodology to assess ventricular scar using custom transmural criteria and a semiautomatic approach to obtain transmural scar maps^8^. This approach can be also implemented in upsampled 3D reconstructions^9^ obtained from conventional 2D LGE-CMR sequences. This methodology might provide an intermediate and time-efficient approach to improve performance of 2D LGE-CMR sequences to characterize the myocardial substrate particularly associated with clinical presentation of ICM-related VT episodes and potential recurrences after ablation. This may be especially relevant in patients without primary prevention indication for implantable cardioverter defibrillator (ICD).

We hypothesize that transmural scar assessment using 3D-upsampled data from 2D-LGE-CMR sequences will be sufficient and superior to conventional analysis from 2D-LGE images to identify myocardial regions relevant for infarct-related substrate characterization in patients with ICM and VT episodes. Patient-specific scar reconstructions from isotropic 3D-acquired sequences were used as benchmark comparisons.

## Methods

### Study design

**Figure 1:**
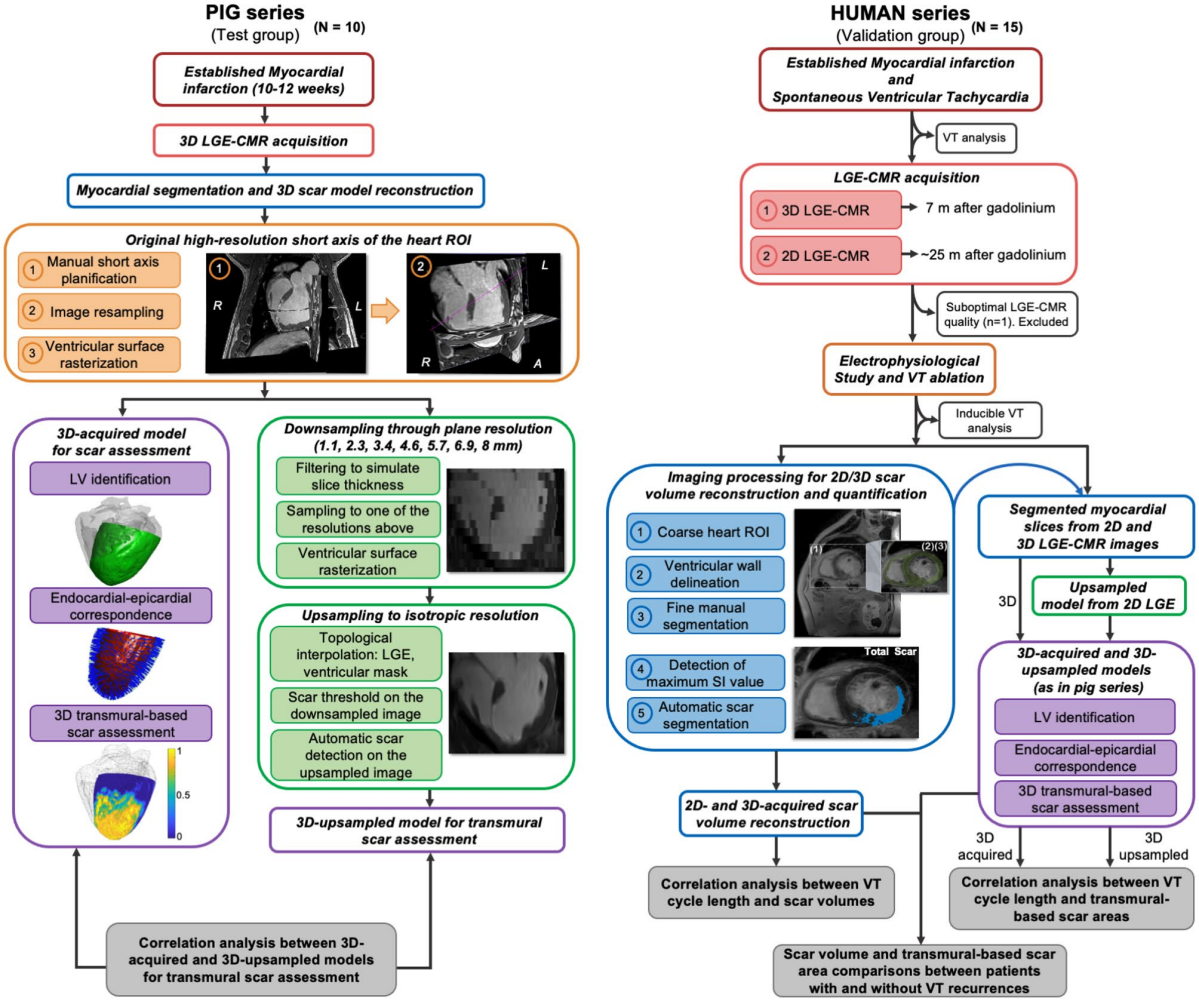
Study flowchart. *LGE-CMR:* late gadolinium-enhanced cardiac magnetic resonance, *ROI:* region of interest, *SI:* signal intensity, *VT:* ventricular tachycardia.

Experimental and clinical study including 10 pigs with established myocardial infarction and 15 patients admitted to hospital with spontaneous VT episodes and underlying infarct-related ventricular scar. All procedures in pigs were approved by the Centro Nacional de Investigaciones Cardiovasculares (CNIC) Committee on Use and Care of Animals and by the Comunidad de Madrid (Ref#PROEX097/17). Animal experiments comply with Spanish (RD53/2013, ECC/566/2015), European (2010/63/EU) and Animal Research: Reporting of In Vivo Experiments (ARRIVE) guidelines. The ethical committees of the Hospital Clínico San Carlos and Hospital Universitario La Paz approved the protocol in patients (Ref#14/246-E_BC and #PI-1464, respectively). The study in patients was performed in accordance with the standards of the 2013 revision of the Declaration of Helsinki. Patients were prospectively included from two tertiary hospitals from May 2013 to December 2016. All patients signed the informed consent before inclusion in the study. Pigs underwent 3D LGE-CMR sequences to test and optimize the performance of a 3D transmural-based methodology that takes into account individual-specific myocardial geometry^8^. The validation and clinical impact of the method were tested in the patients’ cohort undergoing 3D and 2D LGE-CMR studies within the same protocol. Figure 1 shows the study flowchart***.*** The data that support the findings of this study are available from the corresponding authors upon reasonable request.

### Pig model of myocardial infarction

Pigs (male, large-white strain, ~ 35 kg) underwent percutaneous catheterization of the left anterior descending coronary artery using femoral access and fluoroscopic guidance under general anesthesia. An angioplasty balloon was inflated in the coronary artery for 60 min to generate myocardial infarction. Then, the balloon was deflated and a coronary angiogram was recorded to confirm patency of the coronary artery and reperfusion as described elsewhere (see also Supplementary Information)^7^.

### Magnetic resonance studies

Ten-to-twelve weeks after myocardial infarction pigs underwent substrate characterization using a Philips Achieva 3T-Tx whole-body scanner equipped with a 32-element and phased-array cardiac coil (Philips Healthcare, Best, The Netherlands). Seven minutes after intravenous contrast injection (0.2 mM/kg. Dotarem, Guerbet) 3D LGE-CMR sequences were acquired using an inversion-recovery spoiled turbo field echo (IR-T1TFE) with isotropic resolution of 1.5 × 1.5 × 1.5 mm (reconstruction resolution 0.57 × 0.57 × 0.75 mm). Segmented and ECG-gated cine steady-state free precession was also performed to acquire 11–13 contiguous short-axis slices covering the ventricles from the base to the apex to evaluate left ventricular ejection fraction (LVEF) (field of view of 280 × 280 mm; slice thickness of 6 mm without gap; repetition time-TR-2.8 ms; echo time-TE-1.4 ms, flip angle 45; cardiac phases 25; voxel size 1.8 × 1.8 mm; 3 number of excitations).

CMR scans in patients were performed 24-to-48 h prior to invasive electrophysiological procedures for VT mapping and ablation. LVEF was assessed by multiphase-multislice acquisition with a balanced TFE sequence (1.8 × 1.8 × 8.0 mm). Three-dimensional LGE-CMR sequences (acquisition resolution 1.5 × 1.5 × 1.5 mm, reconstruction resolution: 0.70 × 0.70 × 0.75 mm) were obtained based on IR-TFE, seven minutes after intravenous contrast administration. The entire sequence was triggered with a respiratory navigator to compensate for minor volume displacements. The multislice IR-TFE delayed-enhanced 2D sequences (acquisition resolution 1.5 × 1.5 × 8.0 mm, reconstruction resolution: 0.59 × 0.59 × 8.0 mm) were acquired after completion of the 3D sequence. Good scar definition was ensured using a Look-Locker sequence before each acquisition and phase sensitive reconstruction for 3D acquisitions to select correct inversion time for proper healthy myocardium signal nulling.

### Three-dimensional scar volume reconstruction

**Figure 2:**
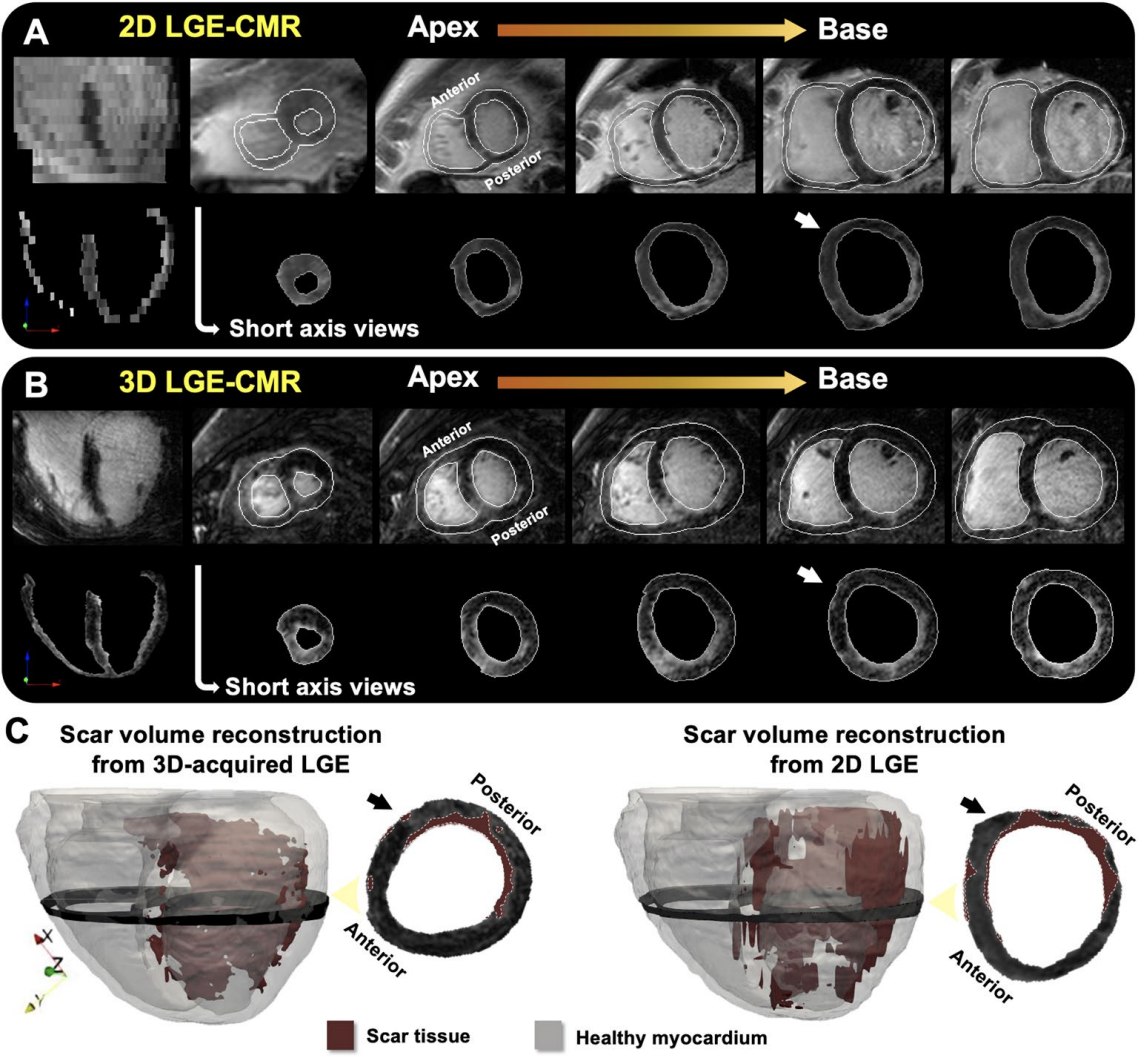
Myocardial segmentation and generation of patient-specific scar-volume reconstructions in 3D models. (**A**,**B**) Identification and segmentation of the endocardium and epicardium of 2D (**A**) and 3D (**B**) LGE-CMR sequences from the same patient after initial automatic processing and fine manual segmentation. (**C**) Representative 3D models with scar volume reconstructions obtained from 2D and 3D LGE-CMR sequences in (**A**), (**B**). Acquisition shifts in 2D slices were corrected using the contours of the 3D-acquired shell.

LGE-CMR sequences, in both pigs and patients, were carefully segmented using a semi-automatic approach with custom-made software in Matlab (Mathworks Inc, Natick, US). First, we obtained a region of interest, which roughly contained the heart and minimized surrounding tissue from other intrathoracic structures. Second, we automatically delineated the endocardial and epicardial contours using an automatic algorithm with an active contour method. Third, this initial myocardial segmentation was subsequently complemented with fine manual segmentation by one single operator and further reviewed by a second expert operator (Fig. 2A,B). This approach, although time-consuming on 3D-acquired sequences (~ 8–12 h) for further implementation in clinical practice, was used as a benchmark reference for highly accurate patient-specific scar volume reconstructions. Finally, scar segmentation was performed using a full-width-half-maximum method^1^. In patients, maximum signal intensity in 3D isotropic acquisitions was calculated using a slice thickness of ≈ 8-mm (i.e. average of 12 neighbouring voxels in the through-plane direction) to prevent any potential signal-to-noise effect from smaller pixel sizes compared to calculations in lower resolution 2D sequences (Supplementary Fig. 1). Scar threshold criterion was set at 0.45 of the maximum signal intensity based on our previous data using high resolution in vivo and ex vivo comparisons^7^. Representative 3D scar-volume reconstructions are shown in Fig. 2C. Further details can be found in the Supplementary Information.

### Three-dimensional transmural-based scar assessment

**Figure 3:**
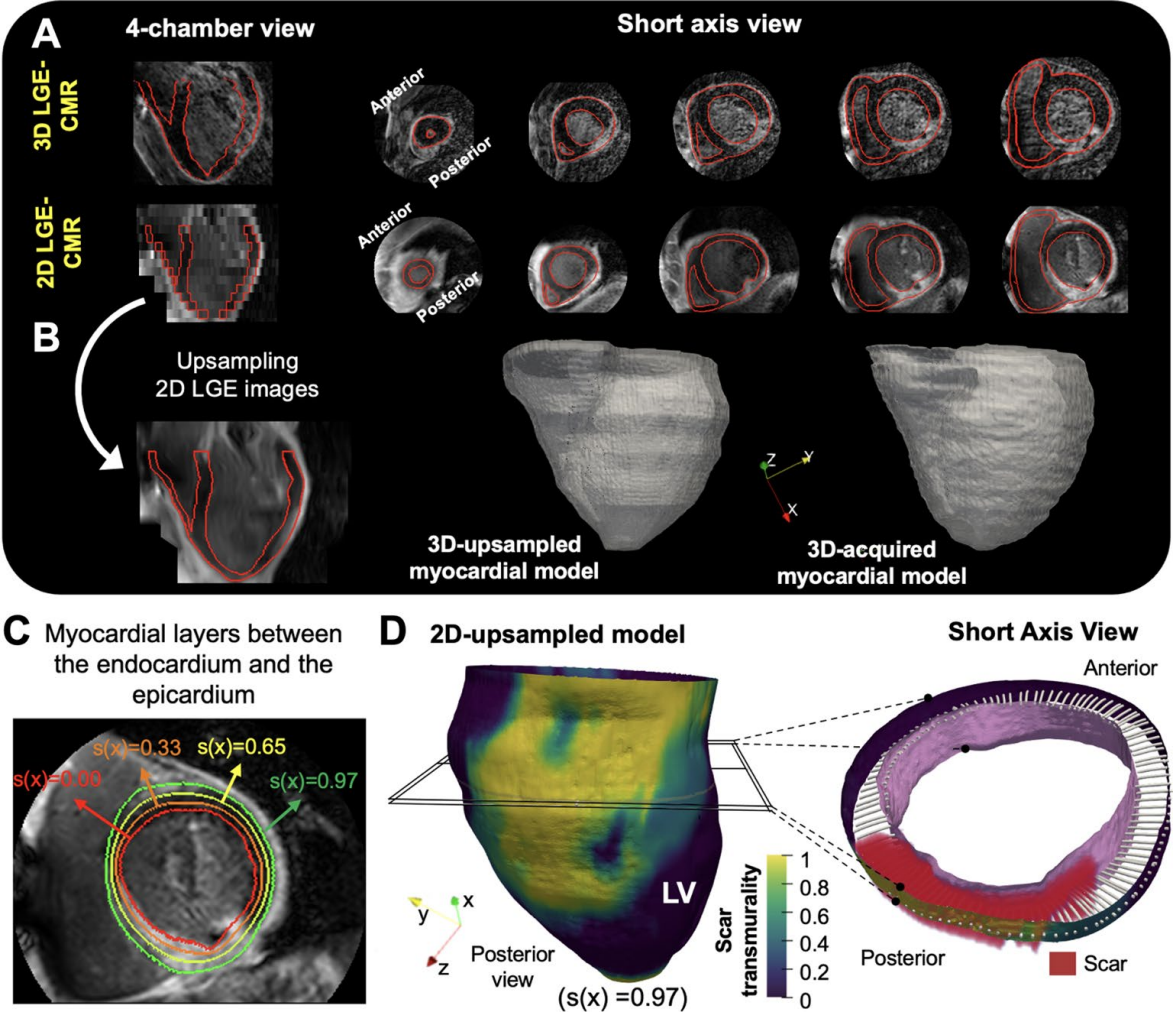
Three-dimensional transmural-based scar assessment. (**A**) Long-axis and short-axis views of 2D and 3D LGE-CMR images from the same patient. (**B**) Resolution upsampling from 2D images and representative 3D-upsampled and 3D-acquired myocardial models. (**C**) Wall segmentation for scar transmurality analysis at different layers. (**D**) Representative transmurality map from a 3D-upsampled model with a short axis view at high-magnification to illustrate the 3D methodology for transmural-based scar assessment. *LV:* left ventricle.

In pigs, 3D LGE-CMR images in the coronal plane were first reoriented in the short axis using 3D Slicer (Slicer 4.10.2) and then reconstructed with different slice thicknesses. For each slice thickness reconstruction, scar segmentations were created by averaging the high-resolution 3D scar segmentations to the new reconstructed slice thickness (Fig. 1). Downsampled images in pigs or 2D LGE-CMR images in patients, together with their associated myocardial segmentations, were upsampled to the 3D isotropic resolution using a topologic interpolation algorithm (Fig. 3A,B) as described elsewhere^9^. Three-dimensional transmurality maps of the left ventricle (i.e., the ratio of myocardial wall thickness covered by scar) were represented on the epicardial surface and computed as reported elsewhere^8^. This method also provides point-wise correspondences between the endocardium and the epicardium (as other authors have done in the past)^10–13^ by finding a function s(x) for which s(x) = 0 at the endocardium, s(x) = 1 at the epicardium, and its intermediate values which define different myocardial layers (Fig. 3C). Transmural-based scar assessment was performed using different transmurality thresholds (0.1, 0.2, etc.) to quantify scar areas (Fig. 3D). Execution times for transmural scar assessment, including point-wise correspondences between the endocardium and the epicardium took on the order of 7 min with unoptimized Matlab code. See Supplementary Methods and Supplementary Figs. 2–4 for further details.

### Mapping and ablation procedure

The invasive electrophysiology study was performed using percutaneous venous and arterial femoral access to reach the right and left ventricles, respectively. Additional epicardial access, if necessary, was achieved using a percutaneous subxiphoid approach. Ventricular geometries were generated using the Carto3 (Biosense Webster, Diamond Bar, CA) or the Ensite NavX (Abbott, St. Paul, MN) electroanatomic mapping system. VT induction was attempted using programmed ventricular stimulation from the right ventricular apex. All stimuli were synchronized with the intrinsic QRS complex. The protocol consisted of two sequential basic drive cycle lengths (CLs) of 10 beats each (S1) at 600 or 400 ms. Each basic drive CL was followed by up to 3 extrastimuli (S2, S3, S4) decremented in CL until reaching refractoriness or a minimum coupling interval of 200 ms. If the arising VT was hemodynamically stable, activation maps and entrainment maneuvers were performed with the ablation catheter or a 20-pole steerable mapping catheter (Pentaray, Biosense Webster) to localize the protected isthmus of the reentrant circuit. Ablation was performed with an open-irrigated-tip catheter (Navistar Thermocool, Biosense Webster or FlexAbility, Abbott). Direct current defibrillation was delivered to restore sinus rhythm in case of ventricular fibrillation or hemodynamic collapse. In the case of hemodynamic instability during mapping of VT morphologies documented on admission (i.e. spontaneous clinical VT), deductive reconstruction of the reentrant circuit and ablation was performed using pace-mapping as described by de Chillou et al.^14^. Hemodynamically instable VT morphologies only documented during the electrophysiological study (non-clinical VTs) were targeted using substrate ablation based on late and fragmented potentials. Local abnormal ventricular activities (LAVA) were not specifically ablated^15^. However, during data analysis, LAVA quantification was performed to further describe the electrophysiological substrate specially in the regions with higher density mapping during baseline sinus rhythm.

### Clinical follow-up and analysis of spontaneous and inducible ventricular tachycardia episodes

Ventricular tachycardia CL was measured on VT tracings documented at the time of hospital admission before attempting pharmacological or electrical cardioversion. The number of induced VT morphologies and the CL of each morphology were quantified for each patient. Twelve out of 15 patients underwent implantable cardioverter defibrillator (ICD) implantation before hospital discharge. Patients underwent annual clinical follow-up to detect VT recurrences of any morphology after new hospital admissions or any sustained VT (episode duration > 30 s or terminated by the ICD) detected on the ICD.

### Statistical analysis

Data are expressed as median and interquartile range for quantitative variables, and number and percentage for qualitative variables. Data normality was assessed with the Shapiro–Wilk test. The Mann–Whitney U test was used for two-group comparisons. The Pearson’s correlation coefficient (r) was used for correlation analysis between scar volumes or transmural-based scar areas and VT CLs. A p < 0.05 was considered statistically significant for differences in group comparisons and for a non-null Pearson correlation coefficient. The intraclass correlation coefficient was also used as a measure of concordance (ICC 2.1 version). All data were analyzed in Graphpad Prism6 (California, US) and custom-made software in Matlab.

## Results

Baseline patient and animal characteristics are shown in Table 1. One patient was excluded due to low CMR imaging quality on both the 3D and 2D sequences. Patients (age 69.3 [65.7, 73.0] years old) showed a median of 16 (12.2, 23.2) years from the infarction to hospital admission. None of the patients had previous history of ventricular arrhythmic events, the LVEF was 40.0% (34.8, 42.3%) and 13 out of 14 patients were in NYHA functional class I. In 11 patients, it was possible to acquire sequential 3D and 2D LGE-CMR images within the same study. In the remaining 3 patients, one patient refused the 2D acquisition after the 3D CMR study and in the other 2 patients, 3D sequences required long acquisition times (> 30 min) which provided suboptimal imaging quality.

**Table 1:** Baseline patient and animal characteristics.

	Pigs (N = 10)	Patients (N = 14)	
**Baseline characteristics**			
Male, n (%)	10 (100.0)	12 (85.7)	
Age, pigs (months) patients (years)	5.0 (4.7, 5.4)	69.3 (65.7, 73.0)	
Weight (kg)	54.7 (53.0, 60.8)	78.8 (66.6, 85.5)	
LVEF (%)	34.7 (32.0, 40.2)	40.0 (34.8, 42.3)	
Hypertension, n (%)	–	13 (92.8)	
Diabetes, n (%)	–	2 (14.3)	
Smoking	–		
Current smoker, n (%)		2 (14.3)	
Former smoker, n (%)		8 (57.1)	
Dyslipidemia, n (%)	–	12 (85.7)	
**Medical treatment**			
ACEis/ARBs, n (%)	–	12 (85.7)	
β-Blockers, n (%)	–	14 (100)	
Antiarrhythmic drugs, n (%)	–	0 (0)	
Antiaggregation, n (%)	–	11 (78.6)	
Oral anticoagulation, n (%)	–	5 (35.7)	
MRAs, n (%)	–	2 (14.3)	
Statins, n (%)	–	13 (92.8)	

LGE-CMR sequences	3D LGE-CMR (n = 10)	3D LGE-CMR (n = 12)	2D LGE-CMR (n = 13)
LGE sequence, n (%)	10 (100.0)	12 (85.7)	13 (92.8)
Acquisition resolution (mm^3^)	1.5 × 1.5 × 1.5	1.5 × 1.5 × 1.5	1.5 × 1.5 × 8.0
Reconstruction resolution (mm^3^)	0.57 × 0.57 × 0.75	0.70 × 0.70 × 0.75	0.59 × 0.59 × 8.0
Acquisition time (min)	12.0 (11.25, 13.75)	22.0 (16.0, 27.5)	7.0 (6.25, 7.75)
**Myocardial/scar volume quantification (cm**^**3**^**)**			
Total myocardial volume	105.8 (98.7, 122.6)	199.5 (172.2, 232.0)	205.2 (178.5, 230.8)
Healthy myocardial volume	85.9 (79.5, 92.6)	174.1 (146.8, 201.9)	174.4 (144.9, 190.0)
Heterogeneous scar volume	17.4 (14.0, 22.9)	17.7 (15.5, 19.2)	23.5 (19.5, 26.6)
Dense volume	5.4 (3.9, 7.8)	8.2 (6.1, 9.2)	14.3 (9.3, 17.7)
Total scar volume	23.3 (17.6, 30.9)	26.3 (22.0, 28.8)	34.4 (30.7, 43.2)
**Electrophysiological data**			
Spontaneous VT cycle length (ms)	–	355.0 (332.5, 393.7)	
Inducible VT cycle length (ms)	–	300.0 (290.0, 338.0)	
Inducible VT morphologies (n)	–	2.0 (1.0, 2.8)	
LAVA/total mapping points (n)	–	149 (96, 250)/359 (198, 656)	
LAVA (%)	–	41.0 (34.5, 43.5%)	

Values are expressed as median and interquartile ranges or n (%), as appropriate.

*ACEis:* angiotensin converting enzyme inhibitors, *ARBs:* angiotensin II receptor blockers, *LAVA:* local abnormal ventricular activities, *LGE-CMR:* delayed gadolinium-enhancement cardiac magnetic resonance, *LVEF:* left ventricular ejection fraction, *MRAs:* mineralocorticoid receptor antagonists, *VT:* ventricular tachycardia.

### Three-dimensional-upsampled models showed significant correlation with the 3D-acquired reference

The maximum downsampling factor used for LGE-CMR 3D sequences in pigs reached 8.0 mm slice thickness on the longitudinal ventricular axis (Supplementary Fig. 5A,B). Quantification of 3D surface distortion showed a maximum distance of 0.16 (0.16, 0.17) mm between the 3D-upsampled model using 8-mm slice thickness and the 3D-acquired reference (Supplementary Fig. 5C). Automatic 3D scar transmurality quantification showed ICC > 0.94 between both (Supplementary Fig. 5D).

**Figure 4:**
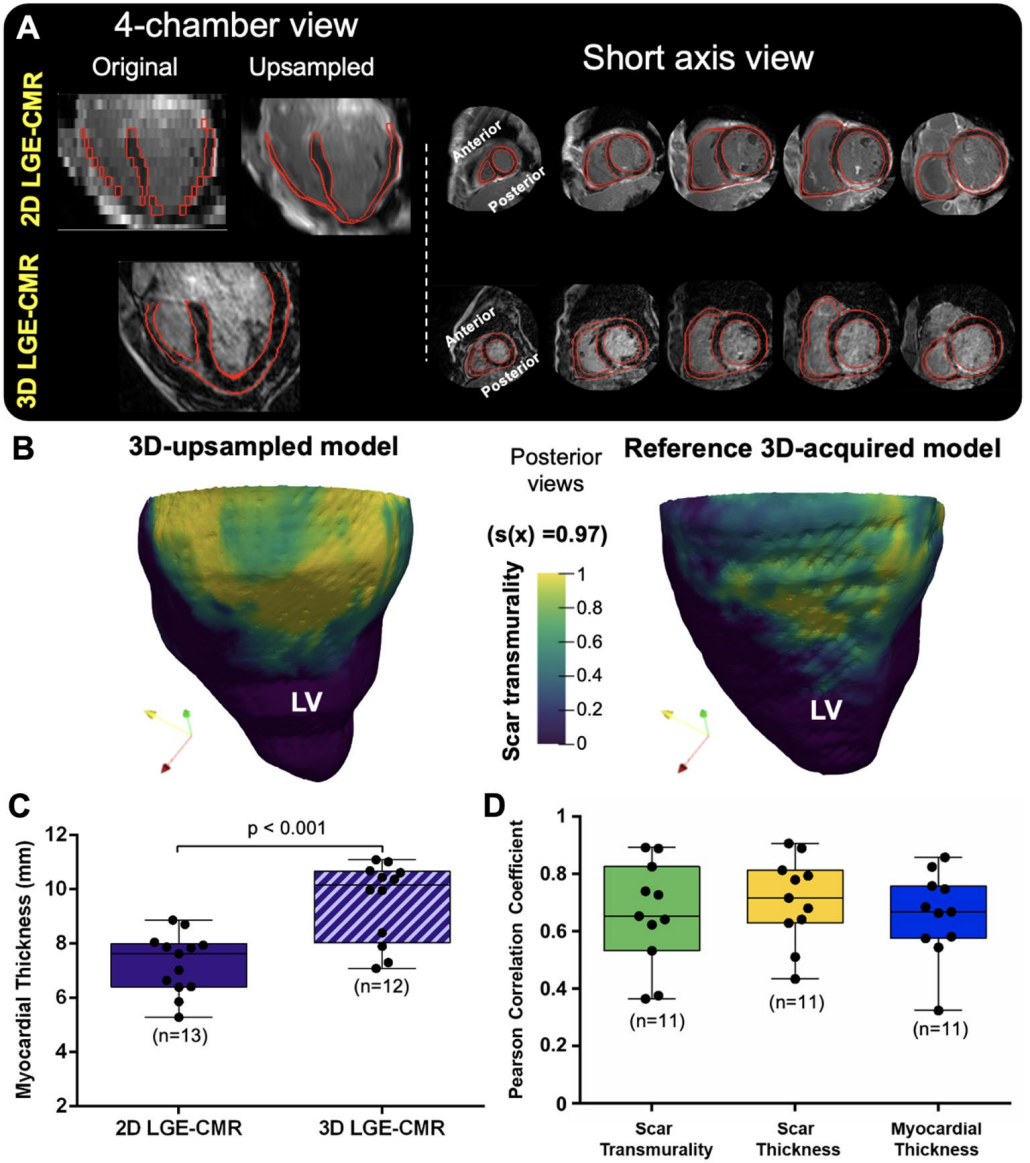
Three-dimensional transmural scar assessment in patients. (**A**) Long axis view of representative 3D and 2D LGE-CMR images from the same patient. (**B**) Sample scar transmurality maps from the 3D and 2D LGE-CMR images showed in (**A**). (**C**) Myocardial thickness comparison between 2D and 3D LGE-CMR images. (**D**) Pearson correlation coefficients of scar transmurality, scar thickness and myocardial thickness between 3D-acquired and 3D-upsampled models in patients. *LV:* left ventricle.

Results in pigs motivated the validation of the methodology using 3D-upsampled models from 2D LGE-CMR images in the clinic (n = 11, Fig. 4A,B). This analysis showed a myocardial distortion of 1.57 (1.46, 2.33) mm from the reference 3D-acquired model. This increase with respect to pigs was mostly explained by different myocardial wall thicknesses in 3D (10.37 [8.03, 10.67] mm) and 2D sequences (7.63 [6.40, 8.02] mm) (Fig. 4C). The Pearson correlation coefficient between transmurality values in 3D-acquired and 3D upsampled 2D-derived models was 0.65 (0.56, 0.81) (Fig. 4D). Analyzing scar and myocardial thickness values separately, the Pearson correlation coefficients were 0.72 (0.63, 0.81) and 0.67 (0.58, 0.76), respectively (Fig. 4D).

### Scar volume quantification showed direct correlation with the cycle length of spontaneous VT episodes

**Figure 5:**
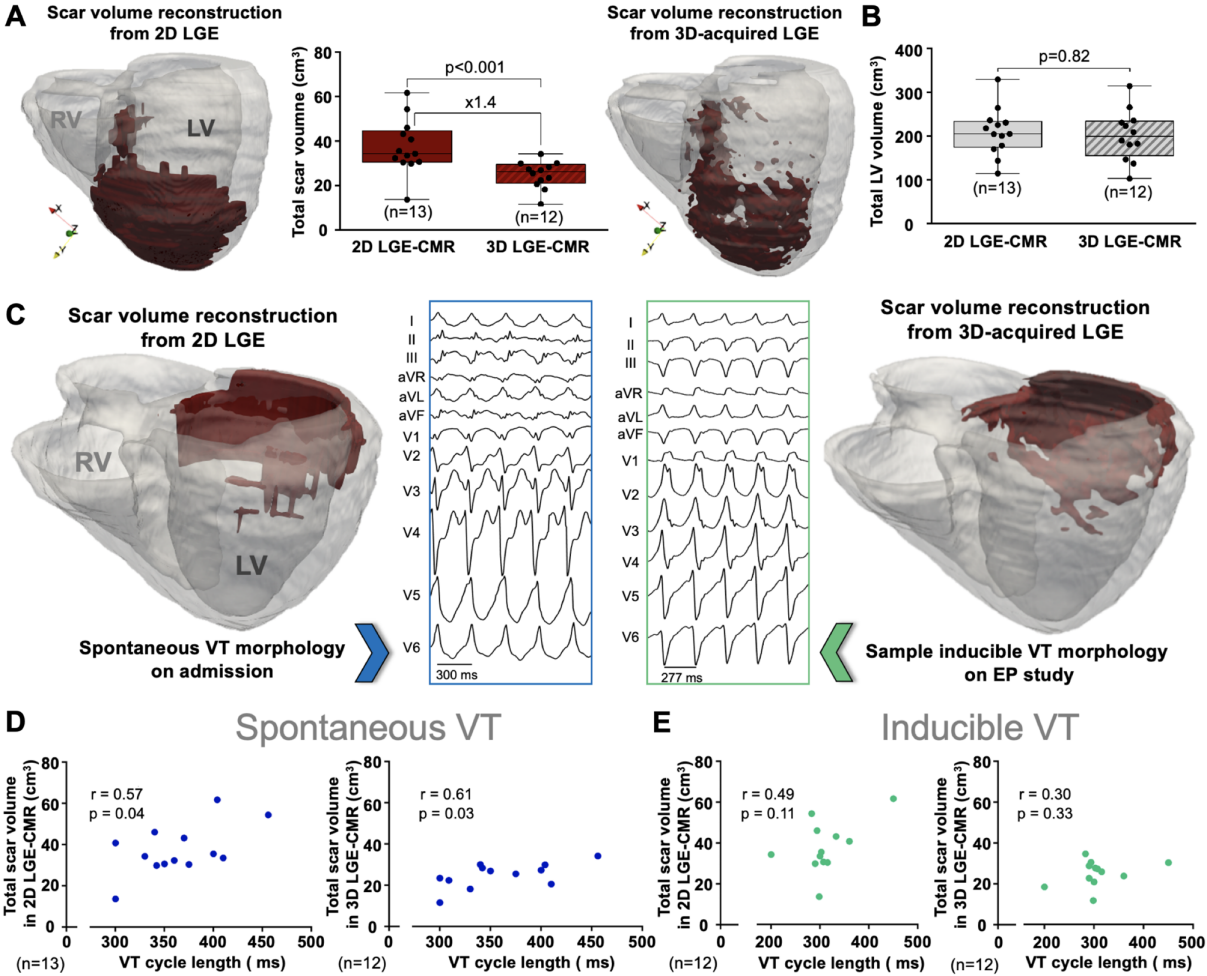
Patient-specific scar volume reconstruction and correlation analysis with the cycle length of ventricular tachycardia episodes. (**A**) Scar volume comparisons between 3D- and 2D-LGE-CMR reconstructions. (**B**) Left ventricular (LV) myocardial volume comparisons between 3D- and 2D-LGE-CMR reconstructions. (**C**) Representative case of 2D- and 3D-LGE-CMR scar reconstructions and the associated ventricular tachycardia (VT) episodes on admission and during the electrophysiological (EP) study. (**D**,**E**) correlation analysis between the cycle length of spontaneous (**D**) /inducible (**E**) VT episodes and scar volumes from 3D (**D**) and 2D-LGE-CMR (**E**) reconstructions. *RV:* right ventricle.

Scar volume quantification from the original 2D LGE-CMR sequences showed larger scar volumes than those from 3D-acquired LGE reconstructions (Table 1, Fig. 5A). Conversely, left ventricular wall volume (including myocardium and scar) was not significantly different between 2D- and 3D-acquired ventricular reconstructions (Fig. 5B). Data from one representative patient who underwent sequential 3D–2D–3D CMR-LGE acquisitions further supported larger scar volume from 2D images compared to scar volumes obtained from any of the 3D acquisitions (Supplementary Fig. 6).

Both 2D- and 3D-acquired scar volume reconstructions (see representative examples in Fig. 5C) showed statistically significant correlation with the CL of spontaneous VT episodes documented at the time of hospital admission (r = 0.57; p = 0.04 and r = 0.61; p = 0.03, respectively. Figure 5D). The larger the scar volume, the slower was the CL of the spontaneous VT episode. More specific analysis using a double cutoff criterion at 0.45 and 0.67 of the maximum signal intensity (for heterogeneous and dense scar characterization, respectively)^7^, showed higher correlation coefficients using 3D-acquired heterogeneous scar volumes (r = 0.72; p = 0.01. Supplementary Fig. 7A). Conversely, dense scar volumes did not show any statistically significant correlation with spontaneous VT CLs (Supplementary Fig. 7B). The median CL of inducible VT episodes per patient did not show statistically significant correlation with 2D- or 3D-acquired total scar volumes (Fig. 5E).

### Transmural-based scar assessment in 3D-upsampled models is sufficient to identify scar regions associated with the cycle length of spontaneous VT episodes

**Figure 6:**
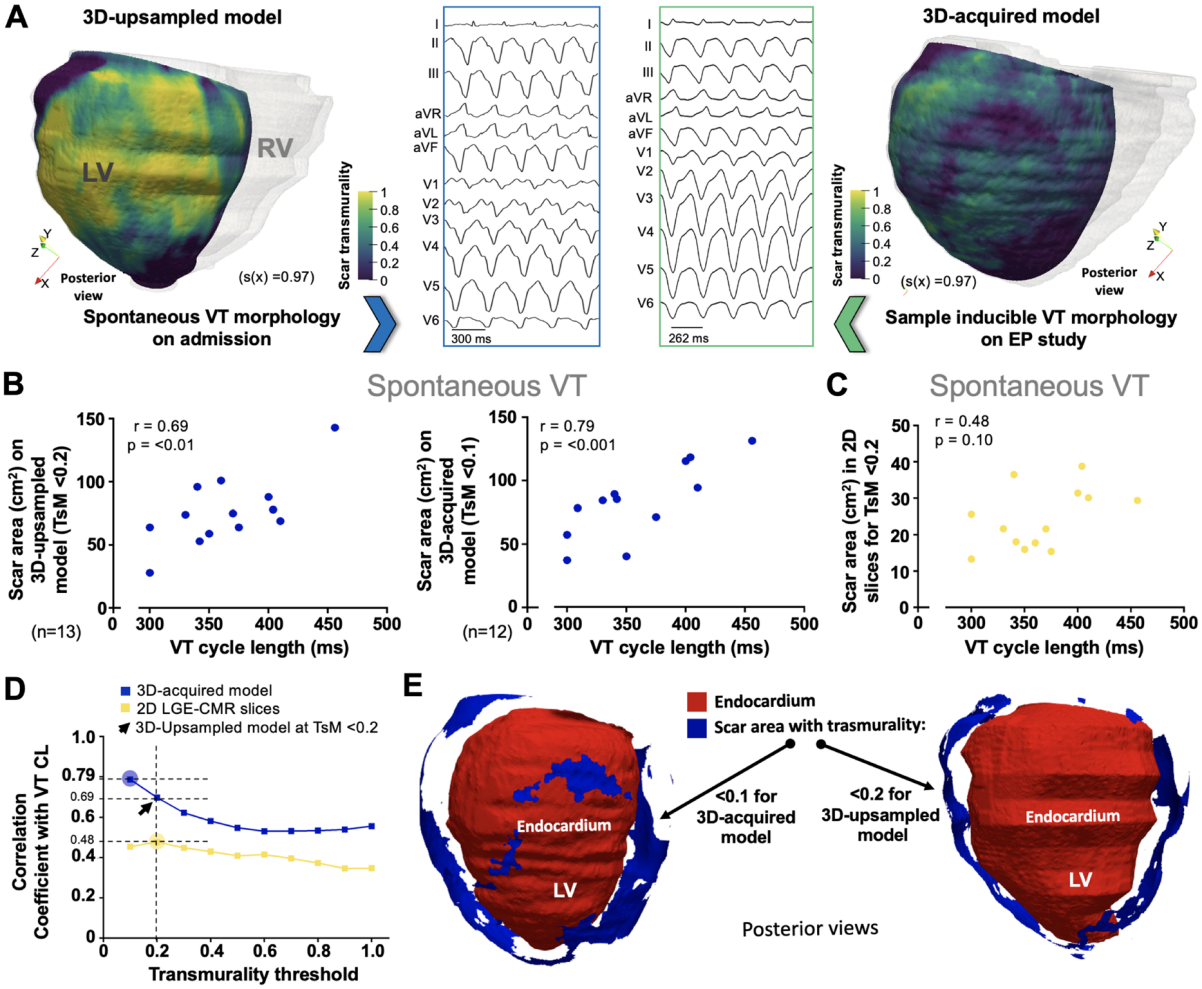
Correlation analysis of transmural scar assessment with the cycle length of ventricular tachycardia episodes. (**A**) Representative 3D-acquired and 3D-upsampled models with transmurality (TsM) maps and the associated ventricular tachycardia (VT) episodes on admission and during the electrophysiological (EP) study. (**B**,**C**) Correlation analysis between the cycle length (CL) of spontaneous VT episodes and scar areas for wall transmurality < 0.2 (for 3D-upsampled models), < 0.1 (for 3D-acquired models) and < 0.2 [for original 2D LGE-CMR slices, in (**C**)]. (**D**) Pearson correlation coefficients between scar assessment at sequential TsM thresholds and the CL of spontaneous VT episodes. (**E**) 3D visualization of scar areas (in blue) using  < 0.2 (for 3D-upsampled models) and < 0.1 (for 3D-acquired models) transmurality criteria in the sample case shown in (**A**).

Scar area quantification in myocardial regions with 3D transmurality < 0.2 for 3D-upsampled and < 0.1 for 3D-acquired models showed significant correlation with the CL of spontaneous VT episodes (r = 0.69; p < 0.01 and r = 0.79; p < 0.01, respectively. Figure 6A,B). The correlation value using transmural scar areas from 3D-upsampled models was higher than the one obtained from scar volume reconstructions from 3D-acquired images (Figs. 6B, 5D, respectively). The transmurality threshold in 3D-upsampled models was selected using the highest correlation coefficient in the original 2D LGE-CMR slices (Fig. 6C,D). In fact, scar areas from 3D-upsampled models showed higher correlation coefficients with spontaneous VT CL episodes than values obtained from conventional 2D transmural scar assessment on the original 2D LGE images (r = 0.48; p = 0.10) using the same transmurality threshold (< 0.2, Fig. 6C). A representative sample case of 3D visualization of scar areas using  < 0.2 (for 3D-upsampled models) and < 0.1 (for 3D-acquired models) transmurality criteria is shown in Fig. 6E. Similar to correlation analysis using scar volume reconstructions (Fig. 5E), the median CL of inducible VT episodes per patient did not show statistically significant correlation with 3D transmural-based scar assessment (Supplementary Fig. 8).

### Transmural-based scar assessment identifies myocardial substrates associated with long-term VT recurrences after ablation

Entrainment maneuvers and pace mapping were performed in 6 and 8 patients, respectively, to target and ablate the myocardial substrate associated with clinical VT morphologies. This and further substrate ablation to eliminate other inducible VT morphologies showed that most of radiofrequency lesions (75 [71, 81] %) were delivered in LAVA regions, although complete LAVA elimination was not pursued in any of the cases. The total ablation time during the procedure was 15.8 (10.1, 17.8) min. Non-inducibility of clinical VT morphologies was achieved in 13 out of 14 cases. In 3 patients, other non-clinical and non-mappable morphologies remained inducible on programmed ventricular stimulation after ablation. Epicardial mapping was only attempted in one patient, although severe pericardial adherences did not enable further mapping of the region of interest and radiofrequency energy was only delivered from the endocardium.

**Figure 7:**
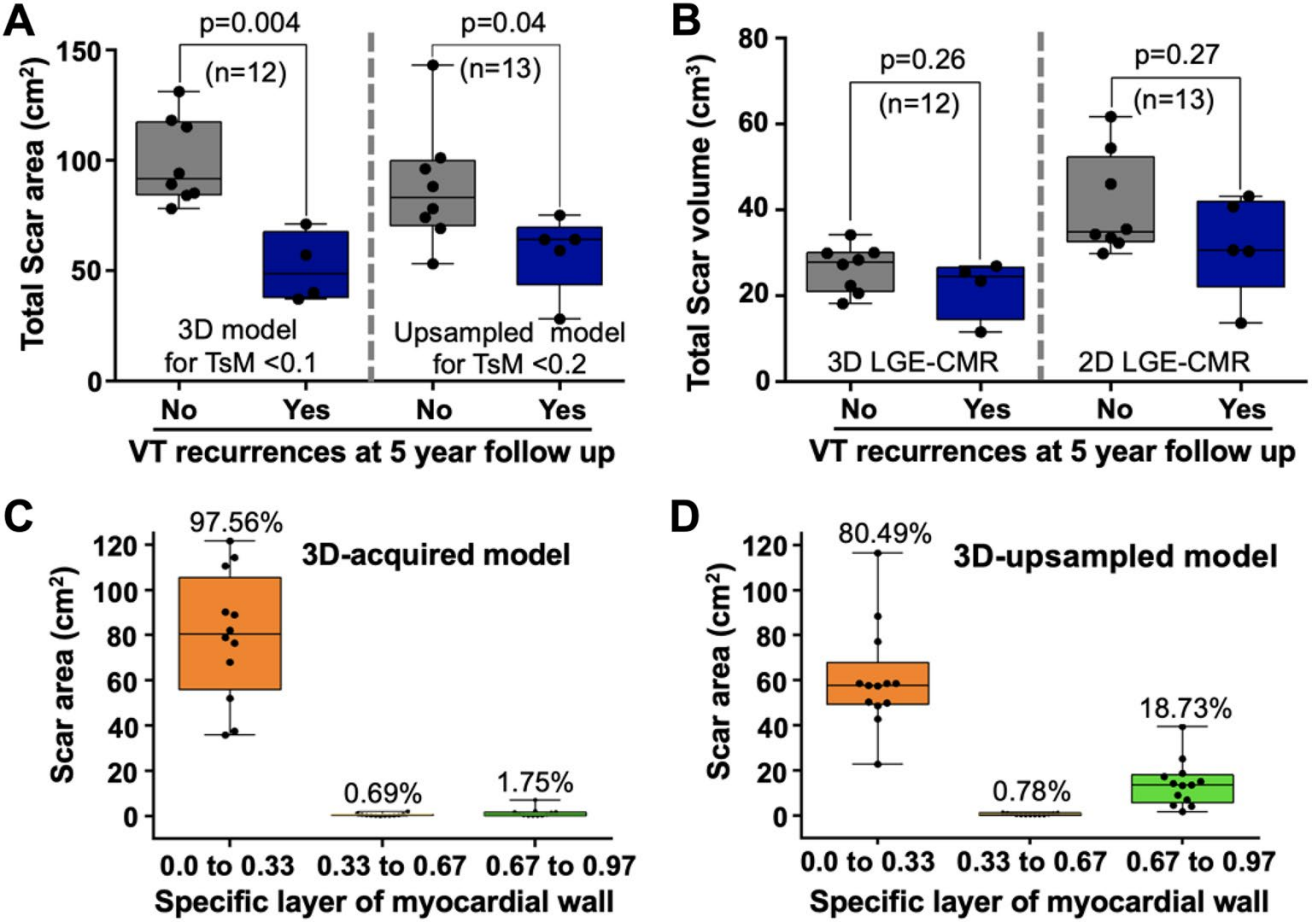
Clinical value of transmural scar assessment in ventricular tachycardia recurrences after long-term follow up. (**A**) Scar areas quantification on 3D-acquired (left) and 3D-upsampled (right) models using < 0.1 and < 0.2 transmurality criteria, respectively, in patients with and without ventricular tachycardia (VT) recurrences. (**B**) Scar volumes quantification from 3D-acquired and 2D LGE-CMR sequences in patients with and without VT recurrences. (**C**,**D**) Scar tissue quantification in the regions with 3D transmurality < 0.1 (for 3D-acquired models) (**C**) and < 0.2 (for 3D-upsampled models) (**D**).

At 5 years of follow-up VT recurrences of any morphology were documented in 5 out of 15 patients. Four patients were under class III antiarrhythmic drugs at the end of the follow-up. Patients with VT recurrences showed lower scar areas on 3D-acquired and 3D-upsampled models using < 0.1 and < 0.2 transmurality criteria, respectively, than patients without VT recurrences (48.5 [37.7, 67.5] vs. 91.5 [84.3, 117.3] cm^2^, respectively, for 3D-acquired models; p = 0.004, and 64.0 [43.5, 69.5] vs. 83.0 [70.2, 99.7] cm^2^, respectively, for 3D-upsampled models; p = 0.04, Fig. 7A). Conversely, scar volume reconstructions from the original images did not reach statistically significant differences between patients with and without VT recurrences (24.5 [14.6, 26.6] vs. 27.9 [21.1, 30.1] cm^3^, respectively, for 3D-acquired LGE-CMR; p = 0.26, and 30.7 [22.1, 42.1] vs. 35.0 [32.7, 52.4] cm^3^, respectively, for 2D LGE-CMR; p = 0.27, Fig. 7B).

Further analysis establishing boundaries in the myocardial wall depth at 0.33, 0.67 and 0.97 of the myocardial thickness showed that the majority of scar tissue in the regions with 3D transmurality < 0.1 (for 3D-acquired models) and < 0.2 (for 3D-upsampled models) was located in the endocardial layer (97.56% and 80.49%, respectively, Fig. 7C,D and Supplementary Figs. 9, 10).

## Discussion

This study shows that semiautomatic transmural scar assessment on 3D models from both 3D-upsampled and 3D-acquired LGE-CMR sequences provide clinically relevant scar characterization in patients with established myocardial infarction. The CL of spontaneous VT episodes significantly correlated with transmural scar values mainly reflecting endocardial scar. Moreover, patients with VT recurrences after a long-term follow-up showed significantly lower scar areas on 3D-upsampled and 3D-acquired models compared with patients without recurrences. Conversely, scar volume quantifications did not reach statistically significant differences between patients with and without VT recurrences. The latter reflects the relevance of endocardial scar assessment rather than the entire scar volume in ICM-related VT episodes. In fact, transmurality criteria at < 0.1 (for 3D-acquired models) and < 0.2 (for 3D-upsampled models), mainly reflected endocardial scar (Fig. 7C,D). Hence, the methodology we propose in this paper may represent an efficient approach in clinical practice after segmentation of myocardial borders and automatic scar detection. Manual accurate segmentation in a small number (≈ 12) of conventional 2D LGE-CMR slices can be performed in less than 15 min by expert operators. However, reliable novel software tools and machine learning algorithms will be a time-efficient alternative for the initial segmentation step^16^.

Several studies have shown that scar tissue quantification using LGE-CMR images may represent an independent predictor of ventricular arrhythmic events after myocardial infarction^3,17^. However, CMR-based scar characterization and quantification to predict ventricular arrhythmic events or VT features remains controversial^18^. Moreover, these associations have not been consistent across studies^19,20^. Imaging resolution, myocardial and scar segmentation, and partial volume averaging represent important factors that may explain the lack of uniform results and variable scar identification criteria among series^21,22^. Partial volume averaging may also explain the differences we documented in myocardial wall thickness using 2D and 3D LGE-CMR images. Larger scar extension is expected to be present in lower resolution images^7,21^, which might substantially affect the predictive performance of scar assessment from LGE-CMR sequences. In fact, our results are consistent with larger scar volumes in patient-specific 3D models obtained from 2D LGE-CMR sequences compared to those from 3D LGE-CMR studies (Fig. 5A). This may explain higher correlation coefficients between the CL of spontaneous VT episodes and scar volumes obtained from higher-resolution 3D-acquired sequences. However, 3D sequences require larger acquisition times compared to 2D sequences (Table 1) and time-consuming reliable segmentation on sequences with > 100 slices, which directly affect regular implementation in clinical practice. The proposed methodology based on 3D transmural scar assessment, using a < 0.2 transmurality threshold in 3D-upsampled models from conventional 2D LGE-CMR sequences, may help to overcome these limitations since scar areas provided higher correlation coefficients with the CL of spontaneous VT episodes than reconstructed scar volumes from 3D-acquired LGE sequences. This highlights that the transmural-based approach was able to identify scar regions relevant for ICM-related spontaneous VT episodes. In other myocardial substrates with less relevance of endocardial scars, 3D transmural-based scar assessment would also permit quantifying scar using different myocardial layer criteria.

A relevant limitation for further implementation of CMR-LGE sequences as a standard stratification tool in the clinic is the of lack of consensus on a uniform scar evaluation criterion and signal intensity thresholding for scar tissue^6,23^, which varies among series and makes substrate characterization particularly sensitive to sampling bias. This requires additional efforts among the scientific community to reach a more general agreement on scar identification. Here, we used a single signal intensity threshold value to simplify scar tissue differentiation from healthy myocardium. We chose a scar cutoff at 0.45 of the maximum signal intensity based on previous analyses in animal models reporting that below such threshold, in high-resolution postcontrast CMR images, remote myocardial areas started to show false positive scar detection^7^. Although mild fibrotic remodeling may also be present in remote ventricular regions, outside the infarcted region, using a 0.45 criterion on signal intensity we documented that this remote scar was ≤ 1% of the remote myocardium, which is consistent with histopathological data reported in animal models with myocardial infarction^24^. Our results further support that a single 0.45 criterion on signal intensity may be a good reference cutoff for substrate characterization in patients.

Our data provide new insights into the role of scar characterization in clinical presentation and long-term follow-up of patients with infarct-related VT episodes undergoing catheter-based ablation, especially in patients without primary ICD indication (14 out of 15 patients in this series). Lower scar areas in patients with VT recurrences at 5 years of follow-up might reflect more viable myocytes within scar regions^25^, which settles a heterogeneous substrate with areas of potentially slow conduction leading to reentrant ventricular arrhythmias. Two small series by Woie et al*.*^26^ and Alexandre et al*.*^17^ have previously shown that the number of scar islands and scar size, respectively, were associated with the mean CL of VT episodes recorded by ICDs over a segment of 12-to-13 VT intervals from the first non-sustained or sustained monomorphic VT morphology. These short recording periods for analysis have limitations to extrapolate results to longer duration VT episodes or clinically relevant spontaneous episodes admitted to the emergency department. Our current prospective series including patients admitted to hospital with spontaneous VT, without neither previous documented episodes nor ICD, supports the role of LGE-CMR imaging to identify relevant myocardial regions and potentially predict VT features in patients with established myocardial infarction. The results are also consistent with other series suggesting that scar extension is not associated with the mean CL of inducible VT episodes during an electrophysiological study^27^. Similar results were reported in a previous study using a pig model with established myocardial infarction, in which we did not document any statistically significant correlation between total, heterogeneous or dense scar volumes with the median CL of inducible VT episodes after programmed ventricular stimulation^25^. Altogether these results highlight that other factors, beyond the underlying substrate, may be involved in inducible VT episodes (e.g. functional and unstable re-entrant circuits, stimulation site, etc.).

### Limitations

Time-to-imaging acquisition after gadolinium administration was not the same for 3D and 2D LGE-CMR acquisitions. However, in both sequences scar identification was performed using the same spatial resolution and signal intensity criterion. Scar volume results were also consistent with previous data reporting the implications of imaging resolution and partial volume effects on scar assessment^7,21^. Data from one representative patient undergoing sequential 3D-2D-3D LGE-CMR acquisitions also supported the notion that lower resolution images show larger scar regions (Supplementary Fig. 6).

Clinical implications of transmural scar assessment in spontaneous VT features were only studied in patients with established myocardial infarction. Other ventricular events as ventricular fibrillation and polymorphic VT episodes have not been studied in this series, which mainly included patients with mild to moderate decrease in LVEF, without ICD at the time of hospital admission^4^. New data from currently ongoing trials (e.g. CMR-GUIDE trial; NCT01918215) will provide more insight into this specific question. Statistical association of scar areas derived from transmurality analysis with VT recurrences might have been affected by individual specific substrate modification during the ablation procedure. Further series are warranted to confirm these descriptive results in relatively limited ablation strategies without extensive scar homogenization.

## Conclusion

Three-dimensional transmural scar assessment in ventricular models reconstructed from 3D-upsampled or 3D-acquired LGE sequences may provide relevant scar characterization for clinical presentation and long-term ablation outcomes in patients with infarct-related spontaneous VT episodes.

## Supplementary Information

Supplementary Information.

## References

[1] Amado LC (2004). Accurate and objective infarct sizing by contrast-enhanced magnetic resonance imaging in a canine myocardial infarction model. J. Am. Coll. Cardiol..

[2] Disertori M (2016). Myocardial fibrosis assessment by LGE is a powerful predictor of ventricular tachyarrhythmias in ischemic and nonischemic LV dysfunction: A meta-analysis. J. Am. Coll. Cardiol. Imaging.

[3] Klem I (2012). Assessment of myocardial scarring improves risk stratification in patients evaluated for cardiac defibrillator implantation. J. Am. Coll. Cardiol..

[4] Al-Khatib SM (2018). 2017 AHA/ACC/HRS Guideline for management of patients with ventricular arrhythmias and the prevention of sudden cardiac death: Executive summary. Circulation.

[5] Jablonowski R (2017). Cardiovascular magnetic resonance to predict appropriate implantable cardioverter defibrillator therapy in ischemic and nonischemic cardiomyopathy patients using late gadolinium enhancement border zone: Comparison of four analysis methods. Circ. Cardiovasc. Imaging..

[6] Andreu D (2017). Cardiac magnetic resonance-aided scar dechanneling: Influence on acute and long-term outcomes. Heart Rhythm.

[7] Lopez-Yunta M (2019). Implications of bipolar voltage mapping and magnetic resonance imaging resolution in biventricular scar characterization after myocardial infarction. Europace.

[8] Merino-Caviedes S (2014). Multi-stencil streamline fast marching: A general 3-D framework to determine myocardial thickness and transmurality in late enhancement images. IEEE Trans. Med. Imaging.

[9] Cordero-Grande L, Vegas-Sanchez-Ferrero G, Casaseca-de-la-Higuera P, Alberola-Lopez C (2012). A Markov random field approach for topology-preserving registration: Application to object-based tomographic image interpolation. IEEE Trans. Image Process. Publ. IEEE Signal Process. Soc..

[10] Jones SE, Buchbinder BR, Aharon I (2000). Three-dimensional mapping of cortical thickness using Laplace's equation. Hum. Brain Mapp..

[11] Yezzi AJ, Prince JL (2003). An Eulerian PDE approach for computing tissue thickness. IEEE Trans. Med. Imaging.

[12] Prasad M (2010). Quantification of 3D regional myocardial wall thickening from gated magnetic resonance images. J. Magn. Reson. Imaging JMRI.

[13] Khalifa F, Beache GM, Gimel'farb G, Giridharan GA, El-Baz A (2012). Accurate automatic analysis of cardiac cine images. IEEE Trans. Biomed. Eng..

[14] de Chillou C (2014). Localizing the critical isthmus of postinfarct ventricular tachycardia: The value of pace-mapping during sinus rhythm. Heart Rhythm.

[15] Jais P (2012). Elimination of local abnormal ventricular activities: A new end point for substrate modification in patients with scar-related ventricular tachycardia. Circulation.

[16] Leiner T (2019). Machine learning in cardiovascular magnetic resonance: Basic concepts and applications. J. Cardiovasc. Magn. Reson..

[17] Alexandre J (2014). Scar extent as a predictive factor of ventricular tachycardia cycle length after myocardial infarction: Implications for implantable cardioverter-defibrillator programming optimization. Europace.

[18] Santangeli P, Marchlinski FE (2016). Substrate mapping for unstable ventricular tachycardia. Heart Rhythm.

[19] Schmidt A (2007). Infarct tissue heterogeneity by magnetic resonance imaging identifies enhanced cardiac arrhythmia susceptibility in patients with left ventricular dysfunction. Circulation.

[20] Piers SR (2015). Myocardial scar predicts monomorphic ventricular tachycardia but not polymorphic ventricular tachycardia or ventricular fibrillation in nonischemic dilated cardiomyopathy. Heart Rhythm.

[21] Bizino MB (2018). High spatial resolution free-breathing 3D late gadolinium enhancement cardiac magnetic resonance imaging in ischaemic and non-ischaemic cardiomyopathy: Quantitative assessment of scar mass and image quality. Eur. Radiol..

[22] Suinesiaputra A (2015). Quantification of LV function and mass by cardiovascular magnetic resonance: Multi-center variability and consensus contours. J. Cardiovasc. Magn. Reson..

[23] Arenal A (2014). Noninvasive identification of epicardial ventricular tachycardia substrate by magnetic resonance-based signal intensity mapping. Heart Rhythm.

[24] van den Borne SW (2008). Molecular imaging of interstitial alterations in remodeling myocardium after myocardial infarction. J. Am. Coll. Cardiol..

[25] Leon DG (2019). Three-dimensional cardiac fibre disorganization as a novel parameter for ventricular arrhythmia stratification after myocardial infarction. Europace.

[26] Woie L (2011). The heart rate of ventricular tachycardia following an old myocardial infarction is inversely related to the size of scarring. Europace.

[27] Ávila P (2015). Scar extension measured by magnetic resonance-based signal intensity mapping predicts ventricular tachycardia recurrence after substrate ablation in patients with previous myocardial infarction. JACC Clin. Electrophysiol..

