# Supplementary material for: Time-efficient three-dimensional transmural scar assessment provides relevant substrate characterization for ventricular tachycardia features and long-term recurrences in ischemic cardiomyopathy
Source: Sci Rep. 2021 Sep 28;11:18722. doi: 10.1038/s41598-021-97399-w (PMC8476552; doi:10.1038/s41598-021-97399-w)
Supplement: Supplementary file 1 — Supplementary Information. [file 41598_2021_97399_MOESM1_ESM.docx]

**SUPPLEMENTARY MATERIAL**

***Time-efficient Three-dimensional Transmural Scar Assessment Provides Relevant Substrate Characterization for Ventricular Tachycardia features and Long-term Recurrences in Ischemic Cardiomyopathy***

**Supplemental Methods**

**Anesthesia protocol in pigs**

Anesthesia induction was achieved by intramuscular ketamine injection (15 mg/kg), xylazine (2 mg/kg) and midazolam (0.5 mg/kg). Then, the pigs were intubated and mechanically ventilated with oxygen (fraction of inspired O_2_ of 21 %) and anesthesia was maintained with continuous intravenous infusion of ketamine (2 mg/kg/h), xylazine (0.2 mg/kg/h) and midazolam (0.2 mg/kg/h). A continuous infusion of intravenous amiodarone (150 mg/h) was administered during the infarction procedure to decrease the incidence of malignant arrhythmias.

**Infarct model of myocardial infarction**

Pigs underwent percutaneous catheterization of the left anterior descending (LAD) coronary artery to inflate an angioplasty balloon and occlude the artery for 60 minutes. The balloon was inflated either proximal or distal to the first diagonal branch to generate different infarct sizes and variable scar distributions. Unfractionated heparin (300 mg/kg) was also administered at the onset of the instrumentation. The procedure was performed under general anesthesia. If ventricular fibrillation arose during the protocol, non-synchronized shocks were delivered with a biphasic defibrillator. After 60 minutes of occlusion, the balloon was deflated and a coronary angiogram was recorded to confirm patency of the coronary artery and reperfusion.

**Delayed gadolinium-enhanced cardiac magnetic resonance** **Imaging processing for scar volume reconstruction**

Delayed gadolinium-enhanced cardiac magnetic resonance (LGE-CMR) sequences were acquired in a whole body perspective (from neck to abdomen). A preliminary step was to obtain a region of interest (ROI) to avoid surrounding areas outside the myocardial tissue. This process enabled us to simplify cardiac segmentation, since other organs and structures were safely ignored. The initial ROI was achieved using a custom-made MatLab tool (The Mathworks Inc., Natwick). This tool relies on a criterion of intensity values, collecting the three dimensional region of interest of the heart and then applying a morphological erosion filter (i.e. with a cone-shaped kernel) to reject the peripherial tissue outside the myocardium.

After ROI retrieval, a custom-made semiautomatic tool in Matlab enabled us to automatically delineate the myocardial tissue. This algorithm includes the segmentation of the left and right ventricles detecting the epicardial and endocardial borders with an active contour method. Despite this initial step, additonal time-consuming manual segmentation was necessary to achieve fine myocardial segmentation in 3D delayed gadolinium-enhanced cardiac magnetic resonance (LGE-CMR) sequences (~8-12 h in human studies and ~30 h in pig sequences).

**Scar characterization and quantification**

Scar was characterized based on an intensity threshold established at 45% of the voxel maximum signal intensity (SI) value (Total scar = 0.45*maximum SI) based on our previous data reporting that below such threshold, in high-resolution postcontrast CMR images, remote myocardial areas started to show false positive scar detection.^1^ Maximum SI values were calculated taking into account the z-plane resolution for each type of LGE-CMR sequence. More specifically, in patients maximum signal intensity in 3D isotropic acquisitions (through-plane resolution 0.75 mm) was calculated using an average of 12 neighbouring voxels (equivalent to ≈8-mm slice thickness) in the through-plane direction to prevent any potential signal-to-noise effect from smaller pixel sizes compared to calculations in lower resolution 2D sequences (through-plane resolution 8.00 mm) ***(Suppl. Figure 1)***. The latter avoids any resolution-derived bias on obtaining the reference maximum SI***.*** Only pixels within the left ventricular wall and inside the infarct region were considered to calculate the maximum SI. The 0.45 SI threshold was applied to the entire sequence, which enabled us to compute total scar volumes.

**Identification of the left and right ventricular cavities** **in biventricular wall segmentation images**

First, the ventricular cavities were identified on each slice of the biventricular wall segmentation by comparing the slice with the result of a hole filling algorithm (available on Matlab).Then, 3D connected regions were identified by applying a 3D connectivity analysis to the stack of slices of cavities binary masks. The largest and second-largest connected regions are labeled as the left and right ventricular cavities, respectively.

**Generation of the left ventricular epicardial contour**

The purpose of this step was to extract the left ventricular (LV) epicardial contour from the biventricular wall segmentation image. Firtstly, the Laplace equation was solved with boundary conditions (a) ŝ(x) must take the values ŝ(x)=0 at the LV endocardium and (b) ŝ(x)=1 at both the pericardium and the right ventricular (RV) endocardium:

$$\nabla^{2}\hat{s}\left( x \right)=0, subject to \hat{s}\left( \Gamma_{en,LV} \right)=0, \hat{s}\left( \Gamma_{en,RV}\cup\Gamma_{peri} \right)=1$$

This equation was solved using a custom method, similar to the one reported by Gibou et al.^2^.

Then, a rough segmentation of the LV was generated by dilating morphologically the mask ŝ(x)<0.98. All voxels outside the biventricular wall after the dilation were set to zero. This step showed some remaining RV wall at the junction between the LV and the RV (***Suppl. Figure 2A, left panel***). In order to improve the LV mask, we developed a variational framework based on the methodology reported by *Bae et al.*^3^:

$$\min_{u\left( x \right)} \int_{\Omega} \gamma\left( f_{in}\left( x \right) u\left( x \right)+f_{out}\left( x \right)\left( 1-u\left( x \right) \right) \right) dx+2 \int_{\Omega} r\left( x \right)\left| \nabla u\left( x \right) \right|dx$$

where u(x) is an implicit function for the LV (u(x)=0.5 determines the LV epicardium), 0≤u(x)≤1 for all x in the domain, f_in_(x) and f_out_(x) are the local costs of x belonging to the LV mask and being outside the LV mask, respectively, r(x) is the local cost of the variation in u(x) taking place at x and γ>0 is a parameter that controls the relative weight between the fidelity to the data and the solution regularity. f_in_(x) and f_out_(x) are expressed in terms of ŝ(x) and the RV cavity mask, χ_en,RV_(x):

$$f_{in}\left( x \right)=H\left( \hat{s}\left( x \right)-s_{\max,}s_{tol} \right)-\gamma_{gs}H\left( \left| \nabla\hat{s}\left( x \right) \right|- \tau_{lvrv},0.02 \right)$$

$$f_{out}\left( x \right)=H\left( s_{\min}-\hat{s}\left( x \right)s_{tol} \right)-\gamma_{gs}H\left( \tau_{lvrv}-\left| \nabla\hat{s}\left( x \right) \right|,0.02 \right)- \chi_{en,LV}(x)$$

where H(z,s_tol_) is a smooth Heaviside function and s_tol_>0 is a small positive number that controls its smoothness: H(z,s_tol_)=0.5+z/s_tol_+sin(πz/s_tol_)/(2π) if |z|≤s_tol_, H(z, s_tol_)=1 if z>s_tol_ and H(z, s_tol_)=0 if z<-s_tol_. The full expression for the regularity cost function is:

$$r\left( x \right)=\log\left( 1+20 sdist \left( x,\Gamma_{wall} \right)^{2} \right) r_{mid}^{\nu}(x)$$

where sdist(x,Γ _wall_ ) is the signed Euclidean distance from x to the biventricular wall surface Γ_wall_, ν>0 is a parameter and r_mid_(x) has the expression:

$$r_{mid}\left( x \right)=\left( \frac{\sum_{v=1}^{N_{v}} w_{v}\left( x \right)\left( sdist\left( x,\Gamma_{mid} \right)-sdist\left( x_{v},\Gamma_{mid} \right) \right)^{2}}{\sum_{v=1}^{N_{v}} w_{v}\left( x \right)} \right)^{\frac{1}{2}}$$

$$w_{v}\left( x \right)= \frac{1}{{10}^{-4}+ \left\| x-x_{v} \right\|^{2}}$$

where Γ_mid_ is the isosurface ŝ(x)=0.5 and x_v_, v=1,…,N_v_, are the subset of biventricular wall surface triangulation vertices that are located within the rough LV mask. That is, vertices located at points already considered outside of the LV mask do not participate in the computation of r_mid_(x).

Once u(x) was computed, the LV segmentation was generated by dilating morphologically the mask u(x)<=0.5 and setting any voxel outside the biventricular wall to zero. Then, the Laplace equation was solved with new boundary conditions:

$$\nabla^{2}s\left( x \right)=0, subject to s\left( \Gamma_{en,LV} \right)=0, s\left( \Gamma_{ep,LV} \right)=1$$

where Γ_en,LV_ and Γ_ep,LV_ are the endocardial and epicardial LV surfaces. ***Suppl. Figure 2A*** shows the isocontours of ŝ(x) and s(x) on a midventricular slice of a biventricular wall mask. Three-dimensional visualization of this process is shown in ***Suppl. Figure 2B***. ***Suppl. Figure 3*** plots the standard deviation of the Euclidean distances between the isosurfaces ŝ(x)=0.5 and ŝ(x)=s_i_ and the isosurfaces s(x)=0.5 and s(x)=s_i_ for values of s_i_ between 0.6 and 1. The latter show a more homogeneous behaviour, especially for values of s_i_ close to 1, due to our variational procedure.

The function s(x) determines the point to point correspondences between the endocardium and the epicardium implicitly. Every point between the LV endocardium and epicardium belongs to one and only one streamline of s(x). Additionally, isosurfaces of s(x) can be used to establish LV myocardial layers without the need of computing streamlines explicitly. These layers have the advantage of not intersecting each other. To understand this better, ***Suppl. Figure 4A*** shows some streamlines explicitly computed starting from randomly selected points at the LV endocardium of a 3D LGE-CMR sequence, and ***Suppl. Figure 4B***, the isosurfaces s(x)=s_i_, with s_i_=0, 0.33, 0.65, 0.97 of the same volume. Notice that the paired endocardial and epicardial points need not be on the same short axis plane.

**Computation of scar transmurality maps for three-dimensional transmural scar assessment**

Scar transmurality maps were computed on the LV myocardium using the method described in Merino et al.^4^ This method requires both myocardial and scar segmentations, as well as s(x) and the endocardium and the LV masks. The streamlines of s(x) are not explicitly computed by this method. Those streamlines, however, are explictly calculated elsewhere.^5,6^

The resulting scar transmurality maps measure, at every point x in the LV myocardium, the ratio of the myocardial thickness between x and the endocardium that is covered by scar, where thickness is defined as the length of the streamline of s(x) passing through x. When x is located at the epicardium, the value of the scar transmurality map takes into account the full streamline length and is equivalent to the classical definition of scar transmurality.

**Computation of scar transmurality maps on individual 2D LGE-CMR slices**

The scar transmurality map of each 2D LGE-CMR individual slice was computed using a 2D method as follows. Firstly, the LV and RV cavities were identified by applying a hole filling algorithm on the biventricular wall mask slice. Pixels set to one by the hole filling algorithm that were zero in the biventricular wall mask are considered to belong to the LV or RV cavities. On some slices, if no LV cavity could be identified, the rest of the processing steps could not be applied and the slice was discarded. This happened on apical slices with no LV cavity or basal slices where the LV cavity is not fully surrounded by myocardium. On slices with LV cavity, the function ŝ(x) was computed. If there was some RV cavity on the slice, the LV epicardium was generated using the methodology described in section **Generation of the left ventricular epicardial contour**; afterwards, s(x) was computed. If no RV cavity was present, then s(x) = ŝ(x). After this, the slice scar transmurality map was computed using Merino et al.^4^

**Quantification of the area affected by low scar transmurality**

A triangulation of the LV epicardial surface was computed on each available 3D-acquired and 3D-upsampled models. The triangle areas had a median value of 0.20 mm2 (0.08 mm2-0.28 mm2) for the former and 0.13 mm2 (0.05 mm2-0.19 mm2) for the latter. The associated scar transmurality map was sampled at the barycenters of every triangle within the triangulation. The triangle areas where the value of the scar transmurality map, sampled at the barycenter, was greater than zero and less than a certain upper threshold value were added together. This was done for upper threshold values of 0.1, 0.2, …, 1. The Pearson correlation coefficient between the areas and the VTs cycle length was computed

We also evaluated the scar transmurality computed on each individual slice of the 2D LGE-CMR sequences in its original resolution (8 mm). The epicardial contour was divided into segments and the area of the epicardium where the scar transmurality value was greater than zero and less than an upper threshold was computed by multiplying the slice thickness by the length of the epicardial segments that met the criterion for the scar transmurality value. ***Figure 6D*** plots the variation of the Pearson correlation coefficient between the area and the spontaneous VT cycle length with respect to the upper scar transmurality threshold. The maximum correlation coefficient was documented using 0.2 as the upper transmurality threshold.

**Evaluation of similarity between 3D-acquired and 3D-upsampled models**

In pigs, we evaluated the surface distortion between each 3D-acquired model and the 3D-upsampled model generated from each of its downsampled images ***(Suppl. Figure 5)***. The LV epicardial surfaces were obtained as a triangulation of the isosurface s(x)=0.97. Each 3D-derived epicardial triangulation vertex was projected onto the 3D-upsampled model triangles; conversely, each 3D-upsampled epicardial triangulation vertex was projected onto the 3D-derived model triangles. Then, we computed the median of the set of distances between all vertices of both triangulations and their projections.

The point to point similarity between the scar transmurality maps from the 3D-acquired and the 3D-upsampled models was evaluated by, firstly, interpolating the scar transmurality maps on both the 3D-derived epicardial triangulation vertices and on their projections on the 3D-upsampled epicardial triangles. Complementarily, we also interpolated the scar transmurality maps on both the 3D-upsampled epicardial triangulation vertices and on their projections on the 3D-derived epicardial triangles.

In patients with both 2D and 3D LGE-CMR acquisitions, a global rigid registration between both sequences was required to align both acquisitions in the same space. To ensure an adequate identification between vertices and their projections, it was necessary to correct breath hold artifacts by using in-plane translations. We remark that this correction was only applied here for the aforementioned reasons, and it was not used anywhere else in the pipeline. Afterwards, the same methodology previously described for pigs was applied to evaluate surface distortion and point-to-point similarity in scar transmurality and thickness maps of both scar and myocardium.

**Scar distribution evaluation**

In order to evaluate the distribution of the scar at the triangulation subset that fulfills the low transmurality criterion, for each triangle *j* in the subset, Δ^j^, a streamline of s(x), Str^j^, starting at the barycenter of the triangle and ending at the endocardium, was computed using a step length of 0.05 mm. Both the scar segmentation and the s(x) map were linearly interpolated at each segment of the streamline. The full streamline was divided in three intervals, one for each of the myocardial layers established beforehand: 0<s(x)<=0.33, 0.33<s(x)<=0.65 and 0.65<s(x)<=0.97. The scar thickness located within each of these intervals: $T_{S}^{j,1}$, $T_{S}^{j,2}$ and $T_{S}^{j,3}$, respectively, was computed. In order to measure how much of the triangulation subset area was originated by scar within each layer, we defined the area attributed to the layer *i*, A_attr,i_ (i=1,2,3), by adding, for all of the $N_{str}$ triangles in the triangulation subset, the result of multiplying the area A_j_ of each individual triangle Δ^j^$, 1\leq j\leq N_{str},$ by the ratio of its associated streamline scar thickness at interval *i*, $\lambda_{i}^{j}$. That is:

$$A_{attr,i}= \sum_{j=1}^{N_{str}} \lambda_{i}^{j} A_{j}, i=1,2,3$$

where $N_{str}$ is the number of triangles in the subset and:

$$\lambda_{1}^{j}=1-\lambda_{2}^{j}- \lambda_{3}^{j}$$

$$\lambda_{2}^{j}=\frac{T_{S}^{j,2}}{max({10}^{-8},T_{S}^{j,1}+T_{S}^{j,2}+T_{S}^{j,3})}$$

$$\lambda_{3}^{j}=\frac{T_{S}^{j,3}}{max({10}^{-8},T_{S}^{j,1}+T_{S}^{j,2}+T_{S}^{j,3})}$$

***Suppl. Figure 9*** shows the areas attributed to each of the layers by the abovementioned methodology of all the epicardial surface affected by a scar transmurality value greater than zero without upper transmurality thresholds on the 3D-acquired and 3D-upsampled models.

We also evaluated the joint distribution of the scar thickness present in the set of streamlines of all the patients where the transmurality criterion was fulfilled. Each streamline Str^j^ was assigned its pair of values ($T_{S}^{j,1}$, $T_{S}^{j,2}+T_{S}^{j,3}$); the first value is the scar thickness present on the subendocardial layer and the second one is the scar thickness present on the remaining two layers. Then, a bivariate histogram was computed for the 3D-acquired and the 3D-upsampled models, which are shown in ***Suppl. Figure 10***. The normalized correlation coefficient between both histograms was 0.997 and, after excluding the histogram bin at (0,0), the normalized correlation coefficient was 0.828.

**Supplemental References**

1. Lopez-Yunta, M. *et al.* Implications of bipolar voltage mapping and magnetic resonance imaging resolution in biventricular scar characterization after myocardial infarction. *Europace* **21**, 163-174, doi:10.1093/europace/euy192 (2019).

2. Gibou, F., Fedkiw, R. P., Cheng, L.-T. & Kang, M. A Second-Order-Accurate Symmetric Discretization of the Poisson Equation on Irregular Domains. *J. Comput. Phys* **176**, 205-227, doi:10.1006/jcph.2001.6977 (2002).

3. Bae, E., Yuan, J. & Tai, X. Global Minimization for Continuous Multiphase Partitioning Problems Using a Dual Approach. *Int J Comput Vis* **92**, 112-129, doi:10.1007/s11263-010-0406-y (2011).

4. Merino-Caviedes, S. *et al.* Multi-Stencil Streamline Fast Marching: A General 3-D Framework to Determine Myocardial Thickness and Transmurality in Late Enhancement Images. *IEEE transactions on medical imaging* **33**, 23-37, doi:10.1109/TMI.2013.2276765 (2014).

5. Prasad, M. *et al.* Quantification of 3D regional myocardial wall thickening from gated magnetic resonance images. *Journal of magnetic resonance imaging : JMRI* **31**, 317-327, doi:10.1002/jmri.22033 (2010).

6. Khalifa, F., Beache, G. M., Gimel'farb, G., Giridharan, G. A. & El-Baz, A. Accurate automatic analysis of cardiac cine images. *IEEE Trans Biomed Eng* **59**, 445-455, doi:10.1109/TBME.2011.2174235 (2012).

**Supplemental Figures and Legends**


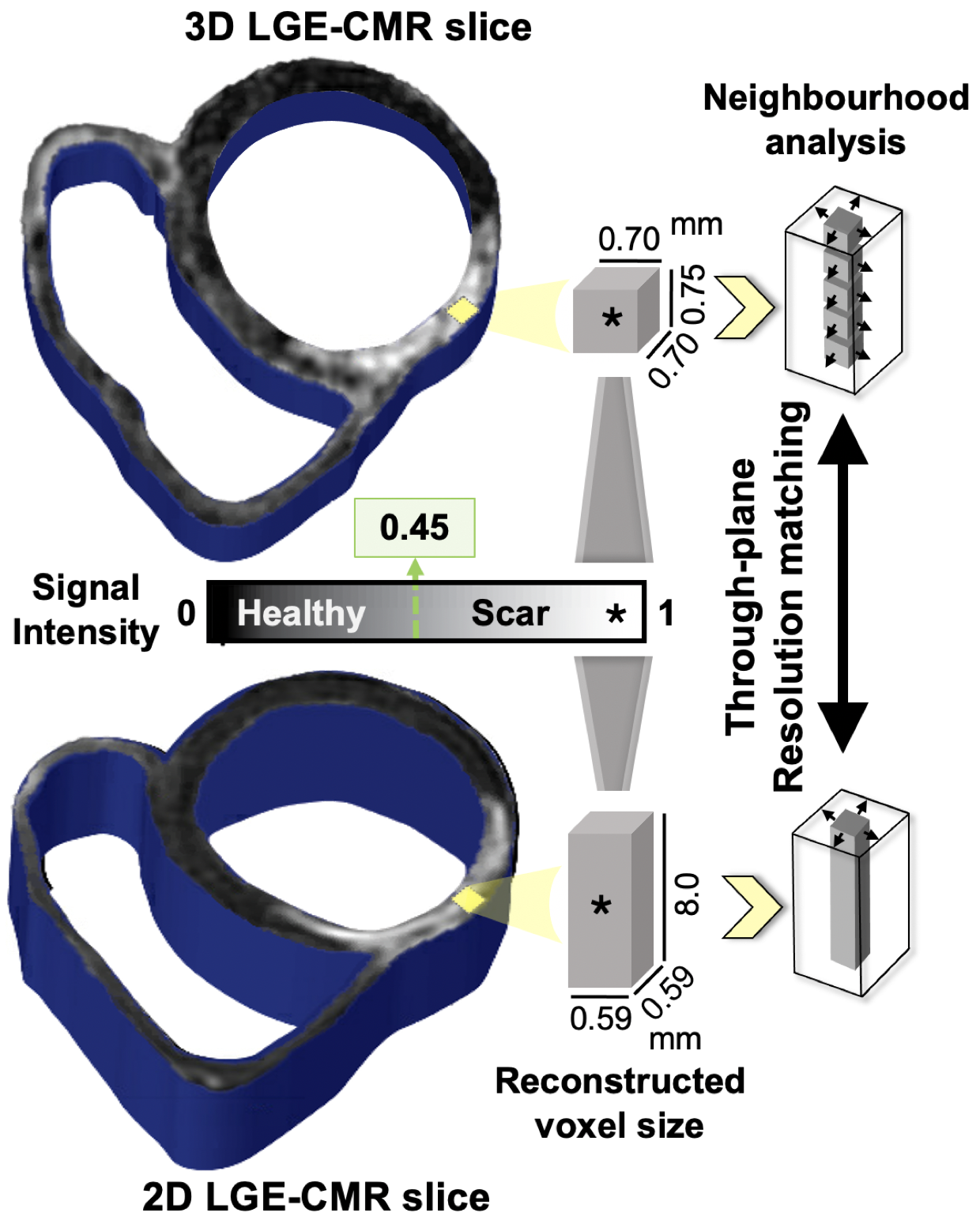


***Suppl. Figure 1.*** Schematic representation of scar segmentation criteria in 2D and 3D images using a full-width-half-maximum method.

***
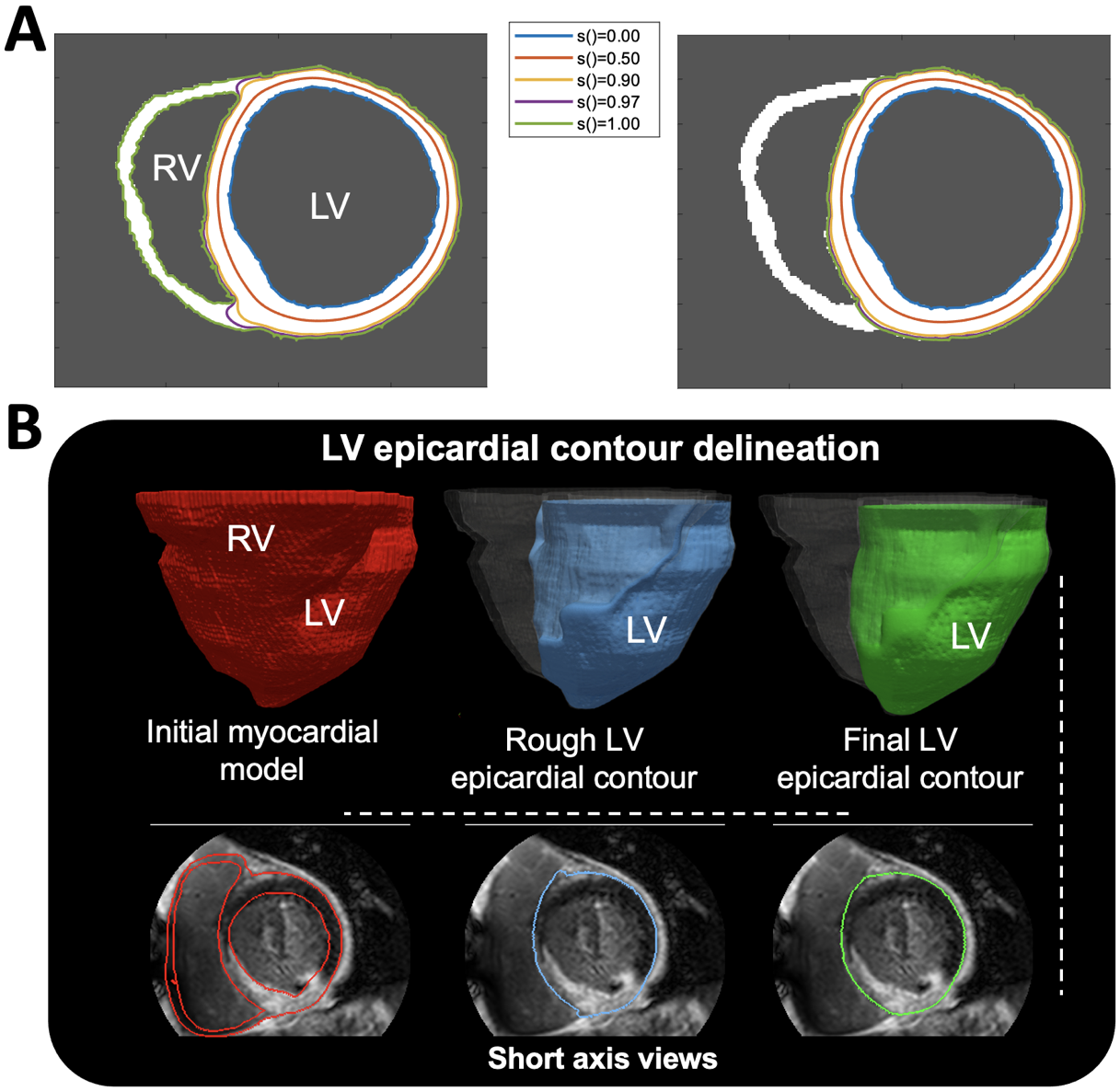
***

***Suppl. Figure 2***. **A,** Isocontours of ŝ(x) (left panel) and s(x) (right panel) on a midventricular slice of a biventricular wall mask on a delayed gadolinium-enhanced cardiac magnetic resonance image from a patient. **B,** Three-dimensional visulización of left ventricular identification and segmentation from LGE-CMR images.


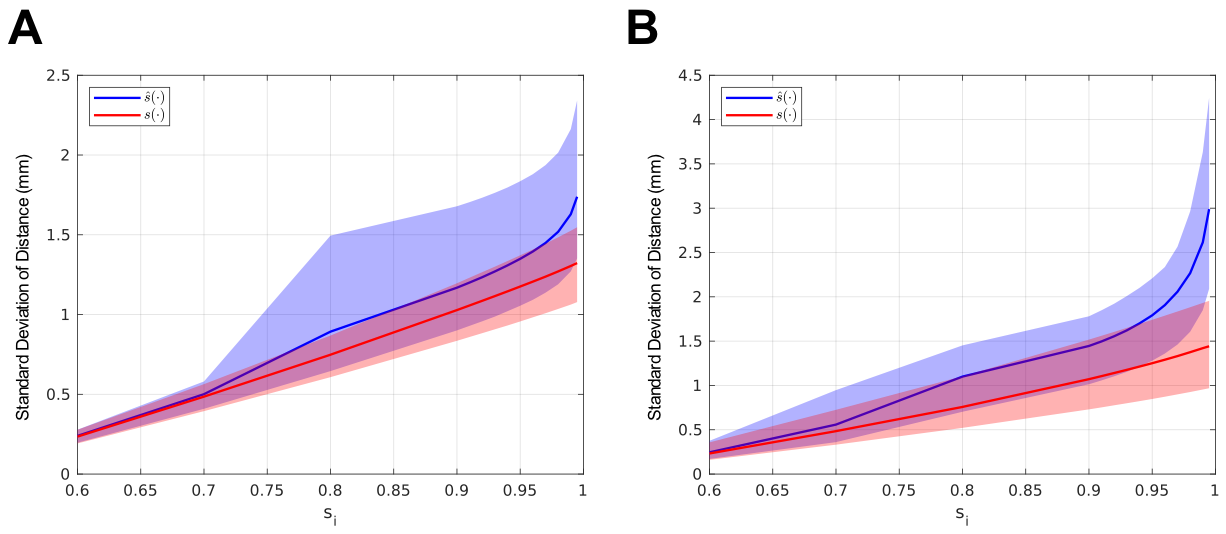
***Suppl. Figure 3.*** Standard deviation of the distance between the surfaces ŝ(x)=0.5 and ŝ(x)=s_i_ (in blue) and the surfaces s(x)=0.5 and s(x)=s_i_ (in red). **A,** Results in pig 3D LGE-CMR sequences (n=10). **B,** Results in patient 3D LGE-CMR sequences (n=12). The continuous line indicates the mean value, and the shadowed region is delimited by the minimum and maximum values.


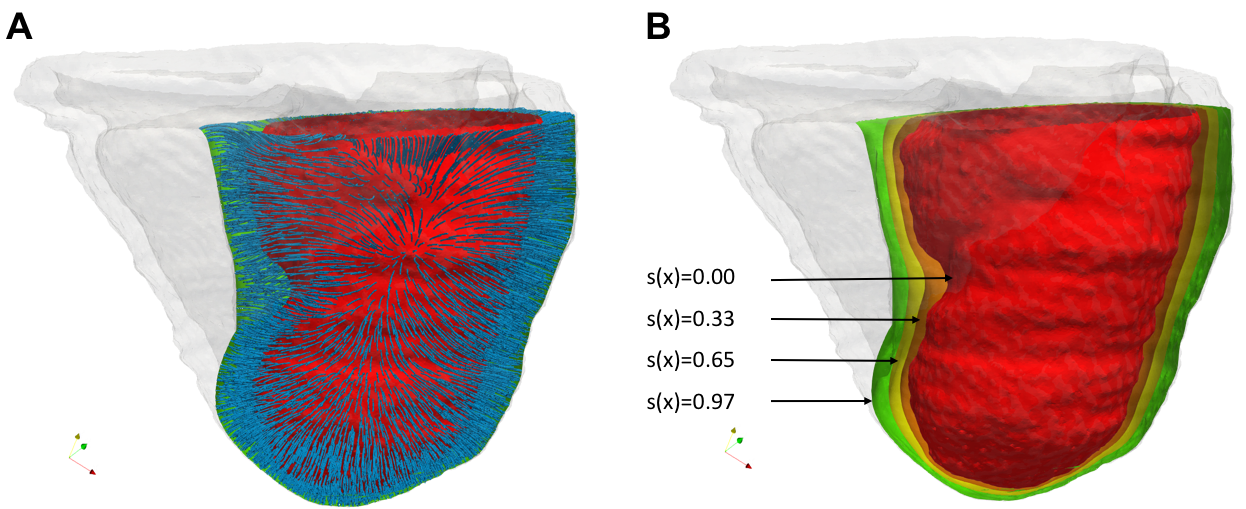


***Suppl. Figure 4***. **A,** Endocardial surface (red) and streamlines of s(x) (blue). **B,** Isosurfaces s(x)=0 (red), s(x)=0.33 (orange), s(x)=0.65 (yellow) and s(x)=1 (green).


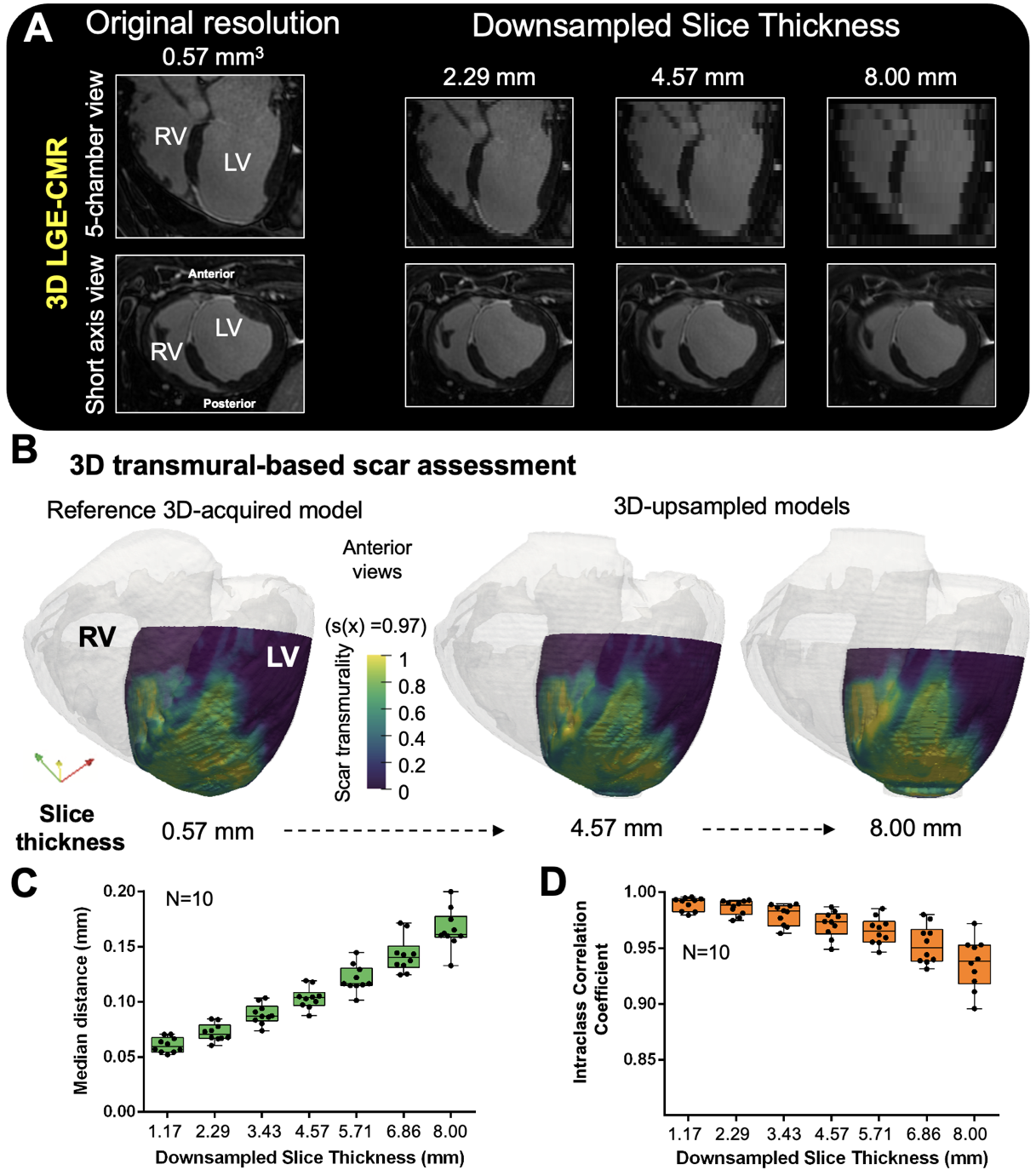


***Suppl. Figure 5***. Three-dimensional transmural scar assessment in pigs. **A,** long axis view of a sample 3D LGE-CMR study and the corresponding downsampled images. **B,** Representative transmurality maps on the reference 3D-acquired model (from the original 3D LGE-CMR sequence) and 3D-upsampled models (from downsampled images). **C,** Correlation analysis of surface distortion between 3D-acquired models and 3D-upsampled counterparts from diferent downsampled images. **D,** Interclass correlation coefficient analysis of 3D transmural scar data between 3D-acquired models (reference) and 3D-upsampled models from diferent downsampled images. LV: left ventricle. RV: right ventricle.


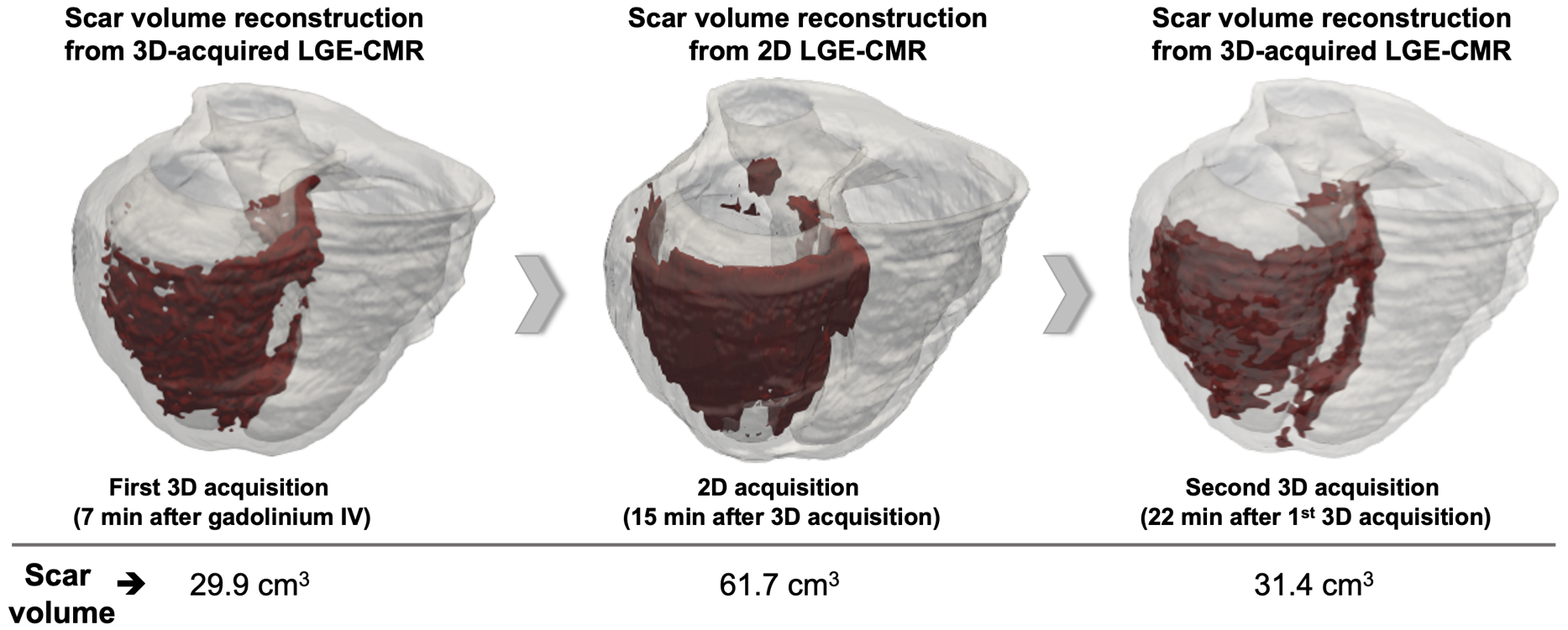


***Suppl. Figure 6***. Three-dimensional reconstruction of scar volumes from sequential acquisitions of 3D-2D-3D delayed gadolinium-enhanced cardiac magnetic resonance (LGE-CMR) sequences in one sample case.


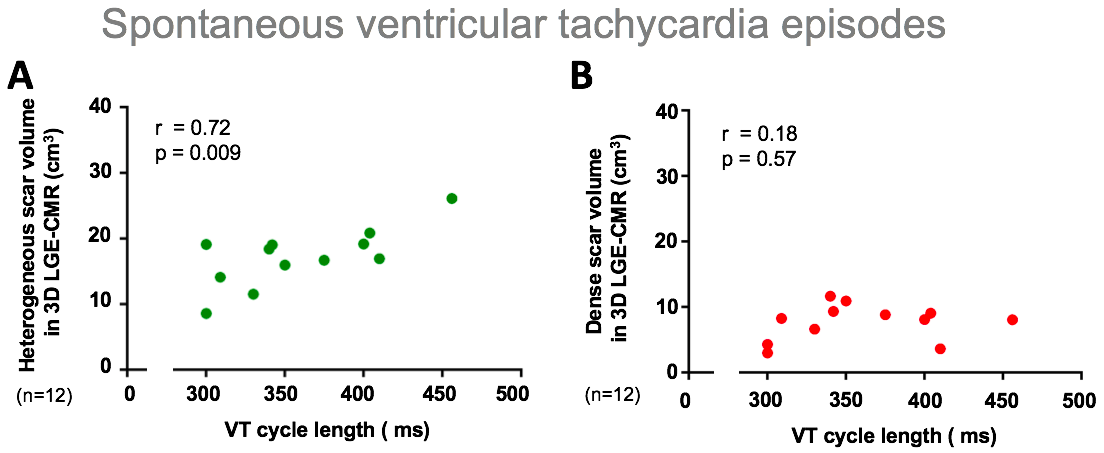


***Suppl. Figure 7***. Correlation analysis between the cycle length of spontaneous ventricular tachycardia (VT) episodes and heterogeneous (A) and dense (B) scar volumes from 3D-acquired delayed gadolinium-enhanced cardiac magnetic resonance (LGE-CMR) sequences


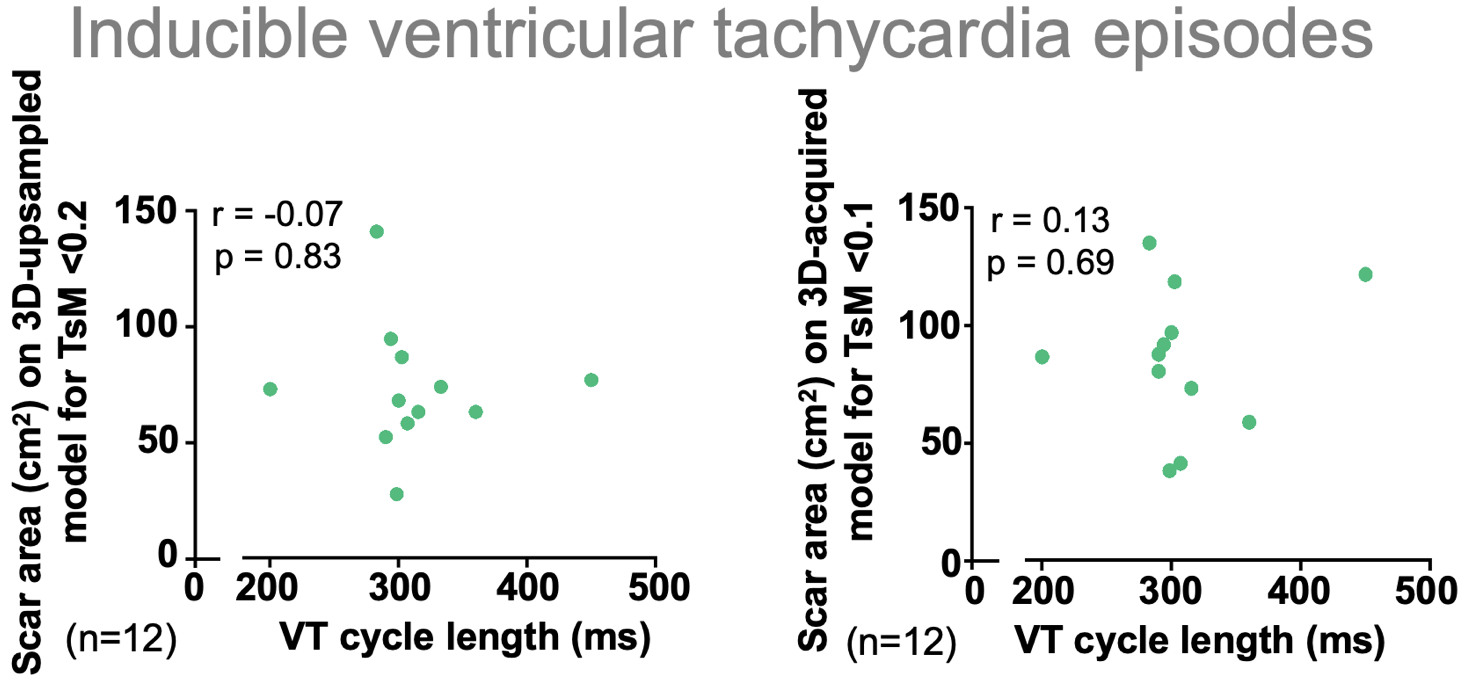


***Suppl. Figure 8***. Correlation analysis between the cycle length of inducible ventricular tachycardia (VT) episodes and scar areas for wall transmurality <0.2 (for 3D-upsampled models) and <0.1 (for 3D-acquired models). TsM: wall transmurality.

***
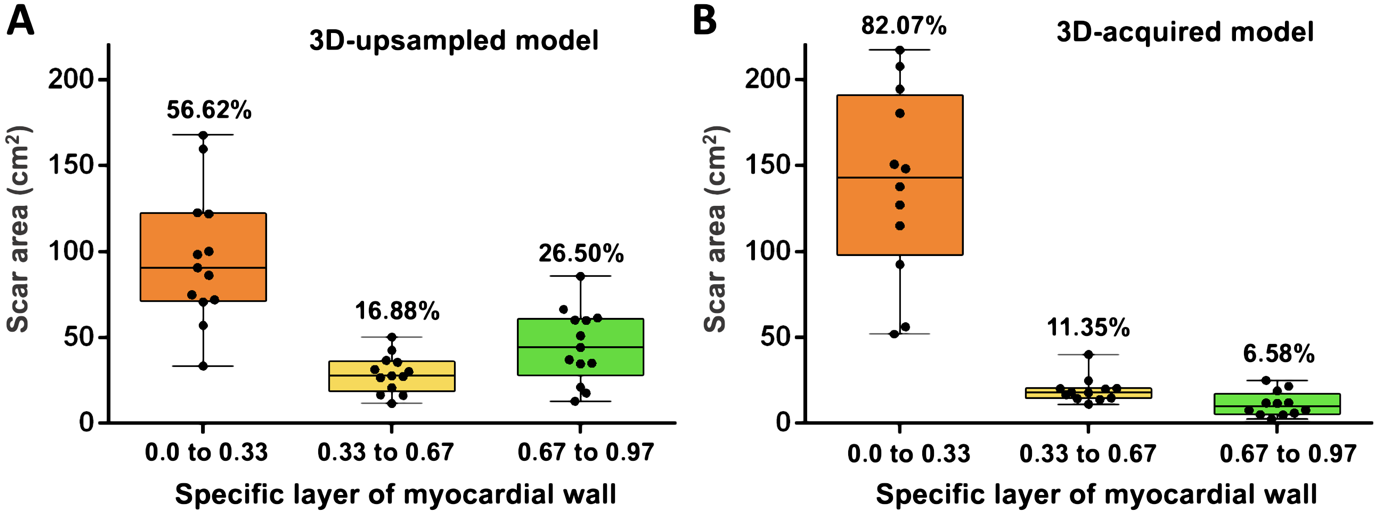
***

***Suppl. Figure 9***. Scar area asigned to each myocardial layer of the epicardial surface with transmurality value greater than zero on 3D-upsampled (A) and 3D-acquired (B) models.

**
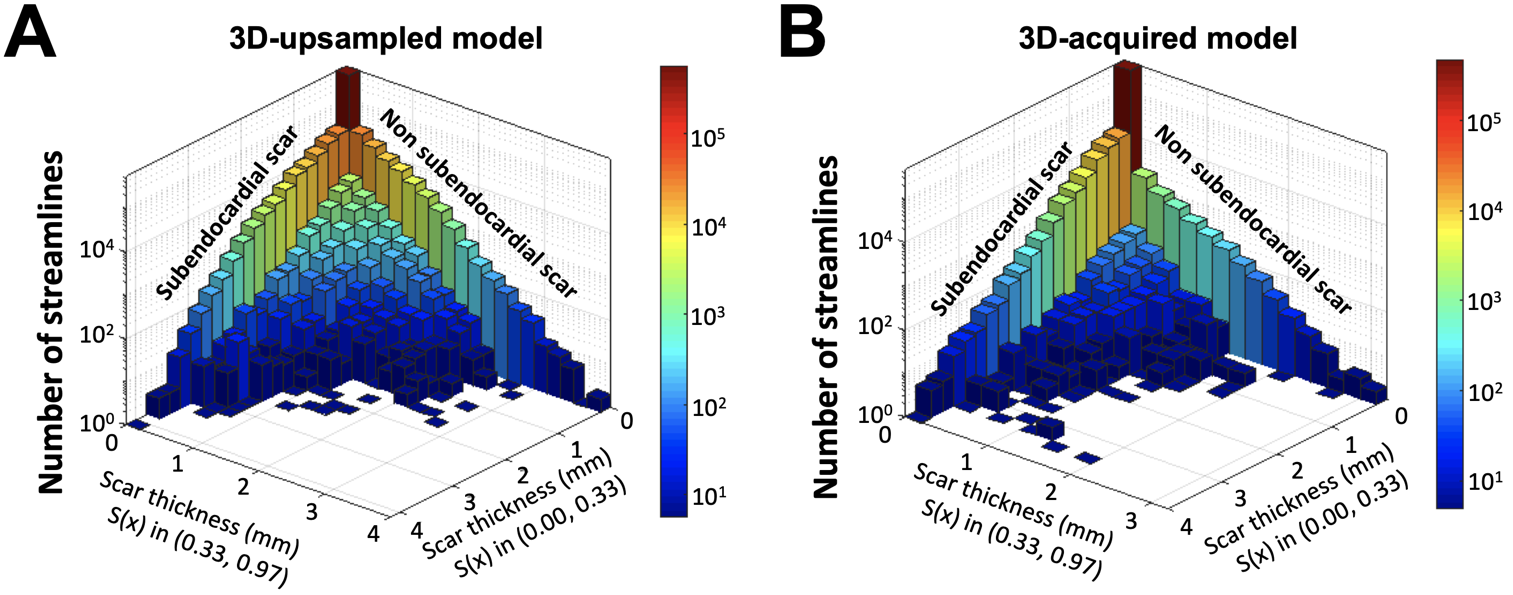
**

***Suppl. Figure 10*.** Quantification of 3D transmural streamlines and scar location in 3D-upsampled (A) and 3D-acquired (B) models.
